# Neutrophil-Derived Protein S100A8/A9 Alters the Platelet Proteome in Acute Myocardial Infarction and Is Associated With Changes in Platelet Reactivity

**DOI:** 10.1161/ATVBAHA.121.317113

**Published:** 2021-11-23

**Authors:** Abhishek Joshi, Lukas E. Schmidt, Sean A. Burnap, Ruifang Lu, Melissa V. Chan, Paul C. Armstrong, Ferheen Baig, Clemens Gutmann, Peter Willeit, Peter Santer, Temo Barwari, Konstantinos Theofilatos, Stefan Kiechl, Johann Willeit, Timothy D. Warner, Anthony Mathur, Manuel Mayr

**Affiliations:** King’s College London British Heart Foundation Centre, School of Cardiovascular Medicine and Sciences, United Kingdom (A.J., L.E.S., S.A.B., R.L., F.B., C.G., T.B., K.T., M.M.).; Department of Cardiology, Barts Heart Centre, St. Bartholomew’s Hospital, London, United Kingdom (A.J., A.M.).; Blizard Institute, Barts and The London School of Medicine and Dentistry, Queen Mary University of London, United Kingdom (M.V.C., P.C.A., T.D.W.).; Department of Neurology, Medical University of Innsbruck, Austria (P.W., S.K., J.W.).; Department of Laboratory Medicine, Bruneck Hospital, Italy (P.S.).; Research Centre on Vascular Ageing and Stroke, Innsbruck, Austria (S.K., J.W.).; Centre for Cardiovascular Medicine and Devices, Queen Mary’s University, London, United Kingdom (A.M.).

**Keywords:** blood platelets, calgranulin A, neutrophils, proteome, ST elevation myocardial infarction

## Abstract

**Objective:**

Platelets are central to acute myocardial infarction (MI). How the platelet proteome is altered during MI is unknown. We sought to describe changes in the platelet proteome during MI and identify corresponding functional consequences.

**Approach and Results:**

Platelets from patients experiencing ST-segment–elevation MI (STEMI) before and 3 days after treatment (n=30) and matched patients with severe stable coronary artery disease before and 3 days after coronary artery bypass grafting (n=25) underwent quantitative proteomic analysis. Elevations in the proteins S100A8 and S100A9 were detected at the time of STEMI compared with stable coronary artery disease (S100A8: FC, 2.00; false discovery rate, 0.05; S100A9: FC, 2.28; false discovery rate, 0.005). During STEMI, only S100A8 mRNA and protein levels were correlated in platelets (*R*=0.46, *P*=0.012). To determine whether de novo protein synthesis occurs, activated platelets were incubated with 13C-labeled amino acids for 24 hours and analyzed by mass spectrometry. No incorporation was confidently detected. Platelet S100A8 and S100A9 was strongly correlated with neutrophil abundance at the time of STEMI. When isolated platelets and neutrophils were coincubated under quiescent and activated conditions, release of S100A8 from neutrophils resulted in uptake of S100A8 by platelets. Neutrophils released S100A8/A9 as free heterodimer, rather than in vesicles or extracellular traps. In the community-based Bruneck study (n=338), plasma S100A8/A9 was inversely associated with platelet reactivity—an effect abrogated by aspirin.

**Conclusions:**

Leukocyte-to-platelet protein transfer may occur in a thromboinflammatory environment such as STEMI. Plasma S100A8/A9 was negatively associated with platelet reactivity. These findings highlight neutrophils as potential modifiers for thrombotic therapies in coronary artery disease.

## Abstract 2

Supplemental Digital Content is available in the text.

**Highlights**

- The first quantitative proteomic study of platelets during acute ST-segment–elevation myocardial infarction demonstrated enrichment of S100A8 and S100A9 protein.
- Synthesis of S100A8 and S100A9 by platelets was ruled out by stable isotope labeling by amino acids in cell culture.
- Neutrophils transfer S100A8 to platelets in free S100A8/A9 heterodimer on coactivation.
- Unexpectedly, plasma S100A8/A9 was associated with reduced platelet reactivity, potentially reflecting in vivo preactivation of platelets in thromboinflammatory states.

Myocardial infarction (MI) and its sequelae remain the primary cause of worldwide mortality and morbidity.^1^ The immediate pharmacological interventions applied in the treatment of MI focus almost exclusively on platelets.^2–5^ Antiplatelet therapy with aspirin has also been conclusively shown to prevent first MI, albeit at the expense of increased risk of bleeding.^6–9^ However, no markers of platelet reactivity have been successfully translated to risk assessment of MI.^10–12^ These clinical observations, and the increasing evidence for alternative pathways of thrombopoiesis,^13,14^ led to the hypothesis that differences in circulating platelets precede and perhaps cause MI.^15,16^

**See accompanying editorial on page 63**

Proteomics approaches, including mass spectrometry (MS), allow interrogation of cellular biology to unprecedented depth.^6^ The platelet proteome has been described, and signaling pathways were identified through phosphoproteomics.^17,18^ Proteomics studies of platelets in cardiovascular disease have predominantly used 2-dimensional gels for resolution of proteins before protein identification by MS.^19–21^ Improvements in multiplexed, tandem mass tag (TMT)-based proteomic analysis,^22^ the availability of stable isotope labeling by amino acids in cell culture (SILAC),^23^ and advances in MS technology offer the opportunity to undertake both more quantitative and dynamic analyses of the platelet proteome. Proteomic studies of platelets in MI have usually been undertaken after the acute illness was treated, percutaneous coronary intervention performed, and dual antiplatelet therapy initiated.^21^ Experimental design to harvest platelets at the immediate time of presentation could significantly enhance understanding of the acute platelet proteome.

The objective of this study was to determine the proteome of platelets during ST-segment–elevation MI (STEMI) and to subsequently investigate proteins that may have a causal role. Protein signatures were interrogated by proteomics using TMT multiplexing, and compared with platelet mRNA levels, as previous studies identified a potential pool of proteins available for synthesis by platelets.^17,24^ The potential for protein synthesis in resting and activated platelets was assessed using a SILAC proteomics approach, and cellular interactions between other innate immune responders were analyzed in clinical and in vitro settings. In the community-based Bruneck study, we evaluated associations with ex vivo platelet reactivity.

## Methods

### Samples From a Clinical Cohort With Acute and Stable Coronary Artery Disease

#### Ethical and Institutional Approval

Clinical samples were collected in this study under specific ethical approval from London–West London & GTAC Research Ethics Committee, REC reference 16/LO/1126, IRAS (Integrated Research Approval System) number 180347. Samples from healthy volunteers were collected under specific ethical approval from the King’s College London Ethics Committee (reference: RESCM-18/19-3676).

#### Case Identification

Participants were recruited to the STEMI arm of the study through active surveillance of the Bart’s Heart Centre Heart Attack Service. Patients undergoing coronary artery bypass grafting (CABG) represent patients with stable coronary artery disease (CAD). These patients were identified through screening of the Bart’s Heart Centre elective surgery lists and selected to match with STEMI patients who had already been recruited. Samples from STEMI patients were drawn on arrival to the emergency center, before the administration of emergency drugs other than 300 mg aspirin and 3 days later. Samples from the CABG group were drawn before and 3 days after surgery. At day 0, no patients had received heparin or P2Y_12_ receptor inhibiting medications. All patients before surgery took low-dose (75 mg) aspirin. Full inclusion and exclusion criteria are available in the Supplemental Methods. In brief, participants were selected for the STEMI group if they were aged between 18 and 80 years, had presented with chest pain and ECG findings of ST-segment elevation, and were subsequently confirmed to have an acutely occluded coronary artery. In the CABG group, patients were selected in the same age range, with angiographically confirmed severe CAD requiring CABG and no history of MI. Participants with other organ dysfunction, hematologic diagnoses, or requiring blood product transfusions in the acute setting were excluded after screening.

#### Preparation of Platelet-Poor Plasma and Washed Platelet Samples

Full details of the sampling and preparation of samples from the clinical cohort are available in the Supplemental Methods. In brief, platelet-poor plasma (PPP) and washed platelets were prepared from samples collected into the acid-citrate-dextrose A buffer, within 2 hours of sampling from the participant. Full blood counts were performed within 15 minutes of blood draw in samples collected into 3.2% citrate buffer, using a Sysmex-2000 FC analyzer.

#### Proteomic Analysis of Platelet Pellets From STEMI and CABG Patients

A full description of proteomic workflow is available in the Supplemental Methods. In brief, all samples from both STEMI and CABG groups, at both time points, were denatured, reduced, alkylated, and digested with trypsin-LysC, then underwent 11-plex TMT tagging and high pH reversed-phase fractionation as per the manufacturer’s instructions. Samples were analyzed using liquid chromatography tandem MS (LC-MS/MS) consisting of a nanoflow UltiMate 3000 high-performance liquid chromatography system (Thermo Scientific) coupled via an EASY-Spray Source (Thermo Scientific) to an Orbitrap Fusion Lumos Tribrid mass spectrometer (Thermo Scientific). Proteome Discoverer software (version 2.2.0.388; Thermo Scientific) was used to search raw data files against a human database (version May 2018, UniProtKB/Swiss-Prot) using Mascot (version 2.6.0; Matrix Science). Tandem mass tags (TMT) were used for quantitative proteomics. Samples from both time points in the same individual were assigned to the same TMT group to allow for robust intraindividual comparisons. STEMI and CABG groups were represented equally in each TMT group to reduce the potential for batch effect bias between experimental groups (Table S1). Raw abundance of each detected protein was scaled to the abundance of the same protein in the pooled sample used in each TMT group and subsequently normalized within each sample.

#### RNA Analysis of Platelet Pellets

Full details of the mRNA analysis of platelet pellets are available in the Supplemental Methods. In brief, RNA was extracted from platelet pellet samples lysed in Qiazol using QIAgen miRNeasy mini kits (catalog No. 217004), following manufacturer’s instructions. cDNA was generated using VILO RT Superscript (Thermo Fisher; catalog No. 11754050) from 8 µL of RNA, again as per manufacturer’s instructions, and transcripts quantified using TaqMan qPCR (quantitative polymerase chain reaction) RNA primers for selected targets, in a ViiA 7 Real-Time PCR System (Applied Biosystems). Relative expression was determined using 2^−^^ddCT^ calculation after normalization using β-actin as an internal housekeeping gene (*ACTB*).

### SILAC Analysis of Platelets, Leukocytes, and MEG-01 Cells

Full details of the isolation, SILAC labeling, and analysis of platelets, leukocytes, and MEG-01 cells are available in the Supplemental Methods. Briefly, washed platelets were isolated from whole blood from 5 healthy adult males. Buffy coats from 2 individuals were collected for leukocyte isolation. MEG-01 cells were cultured as described previously.^25^ Washed platelets, leukocytes from the buffy coat layer, and MEG-01 cells were resuspended in 3 mL of adapted phenol-free RPMI (Roswell Park Memorial Institute Medium) cell culture medium (ThermoFisher; catalog No. A2494201) supplemented with 13C_6_-labeled heavy isotope lysine (Thermo Fisher; catalog No. 89988) and arginine (Thermo Fisher; catalog No. 88210) to a final concentration of 0.274 mM 13C_6_-lysine and 1.15 mM 13C_6_-arginine. Platelets were either lysed after an hour to serve as negative controls (n=5) or rotated and incubated at 37 °C at rest or activation with 30 µmol/L TRAP-6 (thrombin-related activating peptide-6) for 24 hours. Leukocytes and MEG-01 cells were incubated in SILAC medium for 24 hours at 37 °C. Subsequently isolated pellets underwent in-solution digestion, and peptides were analyzed using an LC-MS assembly consisting of a nanoflow UltiMate 3000 high-performance liquid chromatography system (Thermo Scientific) coupled via an EASY-Spray Source (Thermo Scientific) to a Q Exactive HF mass spectrometer (Thermo Scientific), which was configured for data-dependent acquisition using a full MS/data-dependent MS2 setup. Proteome Discoverer software (version 2.2.0.388; Thermo Scientific) was used to search raw data files against a human database (version January 2019; UniProtKB/Swiss-Prot) using Mascot (version 2.6.0; Matrix Science). To detect stable isotope-labeled peptides, the modifications specified in the quantification method (SILAC 2-plex, arginine and lysine residue modification 13C +6.020 Da) were included in the search. Identification of heavy-labeled peptide was first through search of the *m/z* library, then by a semisupervised machine-learning algorithm to generate a confidence (posterior error probability [PEP]) score, and finally manual inspection of spectra.

### Isolation, Activation, and Immunofluorescence of Platelets and Neutrophils

Full details of isolation and immunofluorescence analysis of platelets and neutrophils are available in the Supplemental Methods. In brief, washed platelets were isolated as described previously and neutrophils isolated using Histopaque 1077 (Sigma; catalog No. 10771-500ML) and Dextran gradient purification. For releasate analysis, neutrophils were stimulated with 1 µmol/L N-formyl-methionyl-leucyl-phenylalanine and supernatant collected. Quiescent platelets, mixtures of quiescent neutrophils and platelets, or mixtures of activated neutrophils and platelets supplemented to a final concentration of 2.5 mmol/L calcium chloride and 30 µmol/L TRAP-6 were fixed on microscope slides and incubated with antibodies for CD41 (cluster of differentiation; Abcam; catalog No. ab11024) and S100A8 (Abcam; catalog No. ab92331) and 4′,6-diamidino-2-phenylindole. Confocal microscopy was performed using a Nikon Spinning Disc Confocal Microscope using a 60× oil objective.

### ELISAs

Levels of PPP S100A8/A9 heterodimer were measured using DuoSet ELISA Development kits and Duoset Ancillary Reagent Kit 2 (R&D Systems) as per manufacturers’ instructions.

### High-Performance Liquid Chromatography of Neutrophil Releasate

High-performance size-exclusion chromatography was conducted upon N-formyl-methionyl-leucyl-phenylalanine (1 μM) stimulated neutrophil releasate. Releasate was separated using a Superose 6 Increase 10/300 GL column (Cytiva), 8.6 µm particle size, agarose matrix, and 10 mm internal diameter. One hundred microliters of concentrated releasate was fractionated with PBS as the mobile phase at a flow rate of 0.6 mL/min collecting a total of 36 fractions that were subsequently pooled to generate 12 fractions.

### Immunoblotting

Laemmli sample buffer (4×; 62.5 mmol/L Tris-HCL, pH 6.8, 10% glycerol, 1% SDS, 0.005% bromophenol blue, and 10% 2-mercaptoethanol) was mixed with protein samples and boiled at 95 °C for 10 minutes. Protein samples were separated using either 4% to 12% Bis-Tris (Thermo Scientific) in MOPS (3-(N-morpholino)propanesulfonic acid; Thermo Scientific) at 130 V for 90 minutes. Proteins were transferred onto nitrocellulose membranes in ice-cold transfer buffer (25 mmol/L Tris-base pH 8.3, 192 mmol/L glycine, 20% methanol) at 350 mA for 2 hours. Ponceau S Red staining was used to determine efficient transfer and equal loading before membranes were blocked in 5% fat-free milk powder in PBS containing 0.1% Tween-20 (PBST; Sigma). Membranes were incubated in primary antibodies (S100A9, ab92507; CD9, ab92726; CD63, BD556019; CD81, BD555675; syntenin-1, ab19903) made to appropriate concentrations in 5% BSA in PBST overnight at 4 °C. The membranes were then incubated in the appropriate light chain–specific peroxidase-conjugated secondary antibody in 5% milk/PBST. Membranes were then washed 3× in PBST for 15 minutes. Dot blots were conducted through the blotting of 2 µL of sample directly onto nitrocellulose membrane and allowed to completely dry before following the blocking and antibody incubation protocol outlined above. Western blots and dot blots were developed using enhanced chemiluminescence (GE Healthcare) on photographic films (GE Healthcare).

### PPP and Platelet Reactivity in the Bruneck Cohort

Collection of PPP and measurement of platelet reactivity in the Bruneck cohort have been described previously.^26^ Briefly, 338 participants in this longitudinal study of cardiovascular health donated whole blood for isolation of PPP and light transmission aggregometry (LTA) determining final aggregation using 8 agonists’ concentrations (arachidonic acid, 1 mmol/L; ADP, 5 and 20 μmol/L; collagen, 0.4, 4, and 10 μg/mL; TRAP-6 amide, 25 μmol/L; and U46619, 10 μmol/L). Aspirin use was defined as final aggregation <20% in response to 1 mmol/L per L arachidonic acid in LTA as described previously.^26^ ELISA for S100A8/A9 was performed as above in PPP from these individuals.

### Statistical Analysis

Statistical analyses and figures were produced using the R statistical programming language^27^ (version 3.6.2) and ggplot2. The Shapiro-Wilk test was used to test for normality with a *P* threshold of 0.05. In cases where at least 1 analyte’s values were not normally distributed (eg, in the proteomics comparisons), nonparametric testing applied for all to allow comparison between analytes. In the proteomics comparisons, nonparametric testing was applied including 2-sample Wilcoxon tests (Mann-Whitney *U*) to compare independent groups. Stochastic dominance of an experimental group was determined by Kruskal-Wallis tests and post hoc analysis by Dunn test in selected cases. Parametric testing was applied for the comparisons of the clinical characteristics of the study. Particularly, 1-way ANOVA for all groups and paired *t* test within groups were applied on continuous variables, and χ^2^ tests were applied for categorical variables. Spearman correlation was used for correlations between continuous variables, whereas point-biserial correlation was used for correlations between continuous and categorical variables. The Benjamini-Hochberg correction was used to determine the false discovery rate (FDR) for multiple testing, accepting an FDR threshold of 0.1.

## Results

### Alarmins S100A8/A9 Are Increased in the Platelet Proteome in STEMI

**Figure 1.**
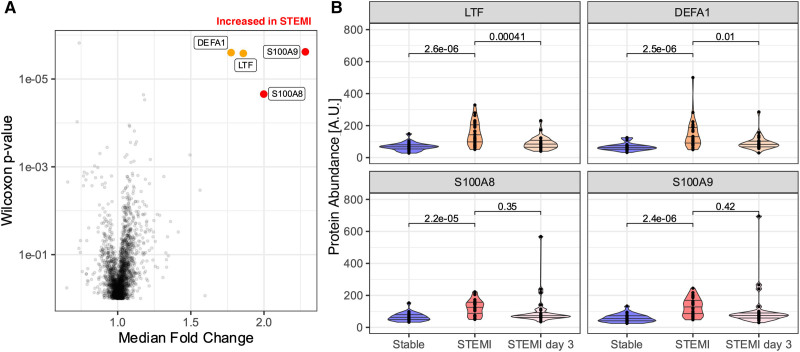
**The platelet proteome in acute ST-segment–elevation myocardial infarction (STEMI).** Washed platelets from 30 individuals experiencing acute STEMI and 25 with severe, stable CAD before CABG surgery underwent proteomic analysis using tandem mass tagged multiplexing. **A**, Volcano plot highlighting proteins with FDR <0.1 and FC of median value ≥1.5 (orange) or ≥2.0 (red) thresholds. **B**, Inflammation-related proteins LTF (lactotransferrin), neutrophil DEFA1 (defensin A1), S100A8, and S100A9 were increased in STEMI patients compared with stable CAD (LTF: FC, 1.85; *P*=2.6×10^−6^; FDR, 0.006; DEFA: FC, 1.77; *P*=2.5×10^−6^; FDR, 0.006; S100A8: FC, 2.00; *P*=2.2×10^−5^; FDR, 0.05; S100A9: FC, 2.28; *P*=2.4×10^−6^; FDR, 0.005).

Washed platelet pellets and PPP from 30 individuals experiencing acute STEMI were sampled on presentation to hospital (day 0) and 3 days later, when clinically recovered. A matched cohort of patients with severe stable CAD awaiting CABG donated washed platelet pellets before (day 0) and 3 days after CABG when the hematologic consequences of cardiopulmonary bypass remain. The Table details clinical and hematologic parameters of both groups. Platelet pellets underwent quantitative proteomic analysis by 11-plex TMT LC-MS/MS. In total, 3570 unique proteins were confidently identified by ≥2 unique peptides. The full list of protein identifications is available in Table S3. Principal component and mRNA analyses confirmed the absence of a TMT group batch effect and differential cellular contamination with white cells (Figures S1 and S2). Two thousand two hundred eighty proteins were identified with high quantitative confidence in every sample. To identify the platelet proteome of patients with acute MI, platelets collected at the time of STEMI were compared with those of patients with stable CAD. A Benjamini-Hochberg FDR <0.1 threshold and fold change ≥2.0 or ≤0.5 was applied. With FDR correction (Figure 1), there were significant increases in S100A8 (FC, 2.00; *P*=2.2×10^−5^; FDR, 0.05) and S100A9 (FC, 2.28; *P*=2.4×10^−^^6^; FDR, 0.005), in patients with STEMI when compared with before CABG. S100A8 and S100A9 varied across both time points and groups (S100A8: *P*=3.80×10^−6^; FDR, 0.009; S100A9: *P*=5.66×10^−7^; FDR, 0.001; Kruskal-Wallis), and post hoc analysis (Dunn) confirmed an interaction between experimental groups at day 0 (STEMI day 0 versus CABG day 0: S100A8, adjusted *P*=2.04×10^−5^; S100A9, adjusted *P*=1.18×10^−6^) and between time points in CABG (CABG day 0 versus CABG day 3: S100A8, adjusted *P*=1.31×10^−4^; S100A9, adjusted *P*=6.1×10^−5^), confirming the relative increase in these proteins during STEMI and post-CABG conditions. Other inflammatory proteins LTF (lactotransferrin) and neutrophil DEFA (defensin A) were increased but did not meet our 2-fold cutoff thresholds for fold change (LTF: FC, 1.85; *P*=2.6×10^−6^; FDR, 0.006; DEFA: FC, 1.77; *P*=2.5×10^−6^; FDR, 0.006). Thus, S100A8 and S100A9 constituted the most pronounced alterations to the platelet proteome during STEMI.

**Table 1.**
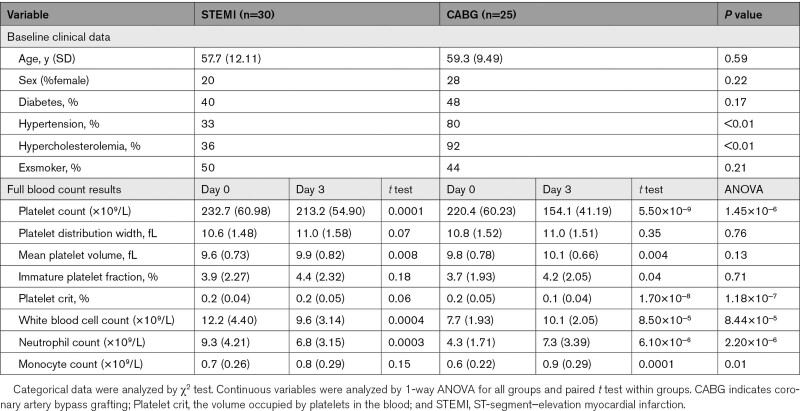
Clinical Characteristics and Full Blood Count Data of the Prospectively Recruited Patient Cohort

### Proteomic Analysis of Platelets Does Not Detect De Novo Protein Synthesis

**Figure 2.**
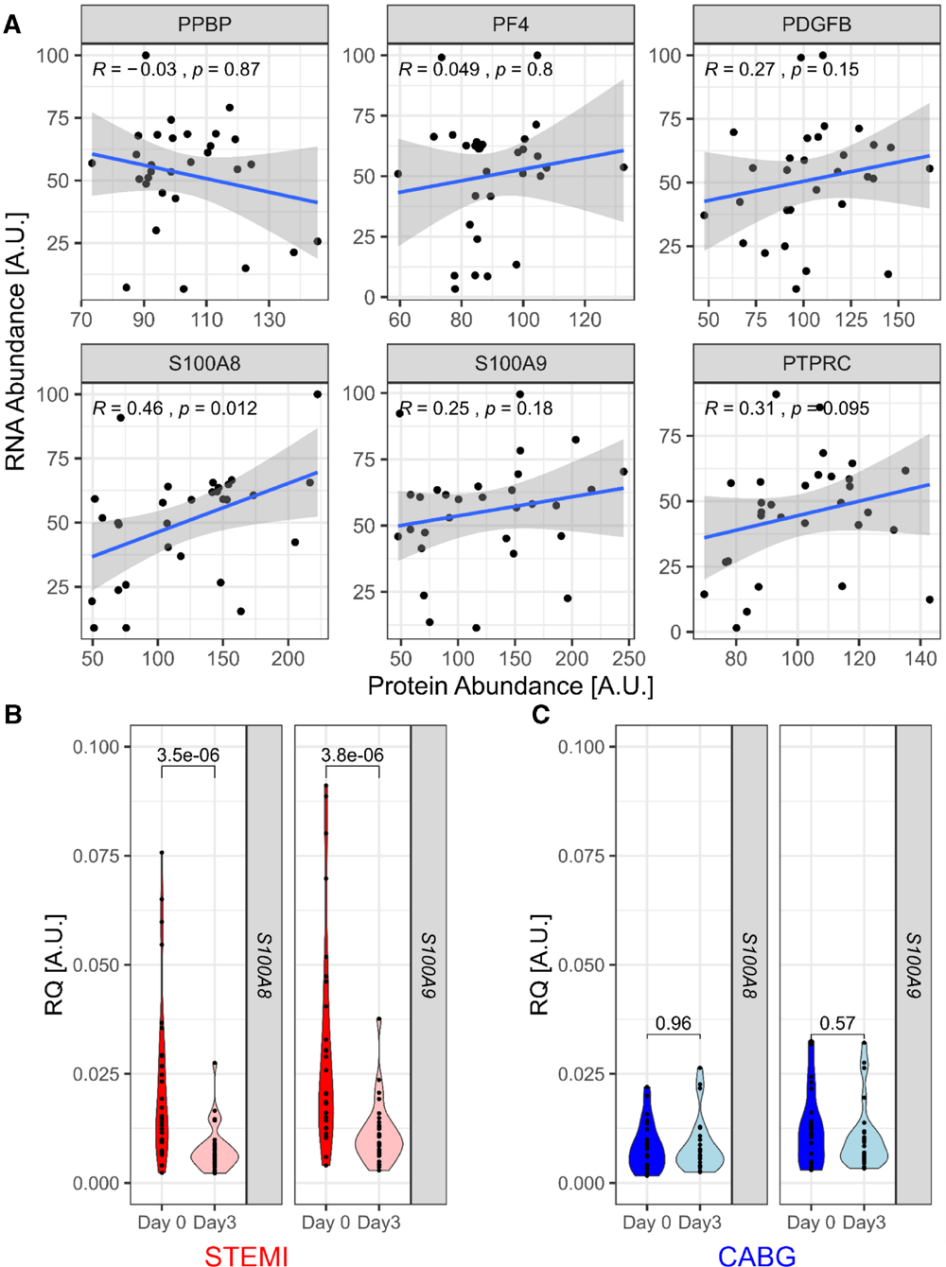
**Platelet S100A8 mRNA is upregulated in ST-segment–elevation myocardial infarction (STEMI).** All washed platelet pellets were analyzed by RT-qPCR (reverse transcription quantitative polymerase chain reaction). **A**, As expected, mRNA and protein abundance for selected proteins did not correlate except for *S100A8*, which demonstrated a moderate, significant positive correlation between mRNA and protein abundance (STEMI day 0, n=30). **B** and **C**, *S100A8* and *S100A9* were significantly reduced 3 d after STEMI but did not change before and after coronary artery bypass grafting (CABG). PDGFB indicates platelet-derived growth factor beta; PF4, platelet factor 4; PPBP, pro-platelet basic protein; PTPRC, protein tyrosine phosphatase receptor type C/CD45; and RQ, relative quantification.

Figure 2A shows the correlation of mRNA and protein abundance of S100A8 and S100A9 in platelet pellets at the time of STEMI. Only S100A8 demonstrates a significant relationship between mRNA and protein abundance (*R*=0.46, *P*=0.012, Spearman, n=30). *S100A8* and *S100A9* mRNA levels were both significantly reduced at day 3 after STEMI (S100A8: FC, 0.42; *P*=3.5×10^−6^; S100A9: FC, 0.45; *P*=3.8×10^−^^5^; Figure 2B), but levels did not change between day 0 and day 3 in the CABG group (Figure 2C). In clinical samples, elevations of mRNA S100A8 and S100A9 appear to be exclusive to the acute STEMI time point.

We, therefore, sought to assess whether changes in the platelet proteome during STEMI can be attributed to de novo protein synthesis by activated platelets. Activated or resting platelets (n=5), leukocytes (n=2), and MEG-01 cells—a megakaryoblastic leukemia cell line—were incubated in SILAC media with heavy-labeled arginine and lysine for 24 hours and then analyzed by LC-MS/MS. The incorporation of heavy-labeled amino acids resulted in a mass shift of the peptides, identifying newly synthesized proteins through detection by MS. The full results of the SILAC analysis are available in Figures S3 and S4 and Table S4. Of a total of >53 000 peptide spectrum matches (PSMs) in the platelet samples, 0.08% (43 PSMs) were identified as incorporating heavy-labeled amino acid, with no differences between resting or activated platelets (activated platelets, 0.10% [18 of 17 305 PSMs]; resting platelets, 0.09% [17 of 18 420 PSMs]; and control platelets, 0.07% [13 of 17 816 labeled PSMs]). PSMs for S100A8 and S100A9 peptides were detected in all platelet samples but not identified as labeled. In highly mitotic MEG-01 cells, 22% of PSMs (5123 of 23 049) and in terminally differentiated leukocytes, 0.35% of PSMs (55 of 15 453) were identified as labeled. Comparison of confidence of PSMs by a semisupervised machine-learning approach (Percolator PEP score) found higher confidence in MEG-01 and leukocyte PSMs than those identified in platelet samples (mean PEP score, 0.009 versus 0.005; *P*<2×10^−16^). Manual inspection of the few heavy-labeled spectra in platelets further found peptide identifications of low confidence, in contrast to examples from MEG-01 and leukocytes, as shown in Figures S3 and S4. Thus, despite confirmation of de novo protein synthesis in cell lines and primary leukocytes, we were unable to provide evidence for de novo protein synthesis in platelets by LC-MS/MS.

### Activated Neutrophils Transfer Alarmins to Platelets

**Figure 3.**
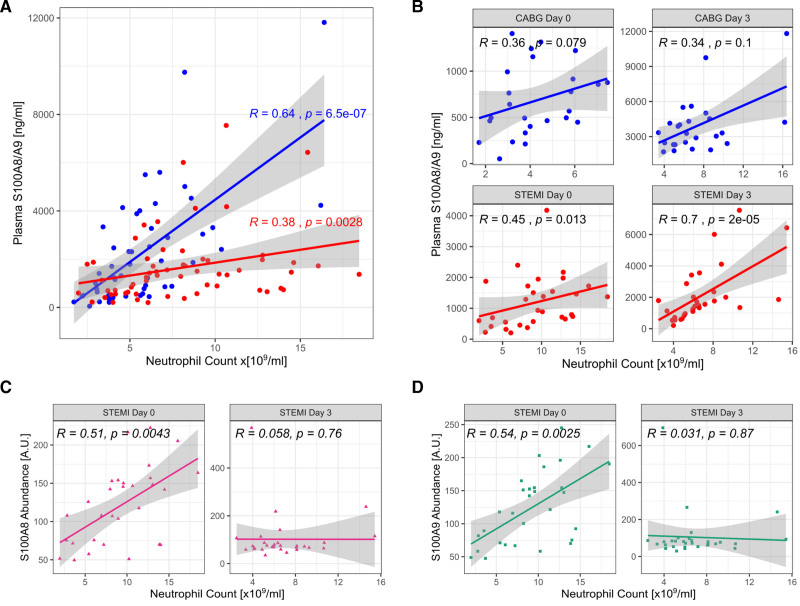
**Abundance of S100A8 and S100A9 in platelets depends on neutrophils. A**, S100A8/A9 is commonly considered to be of neutrophil origin. In patients with stable coronary artery disease (blue), plasma S100A8/A9 positively correlates with neutrophil count. However, this association is less strong in individuals experiencing ST-segment–elevation myocardial infarction (STEMI; red). **B**, When analyzed by time point, the association of plasma S100A8/A9 with neutrophil count is seen primarily at day 3 after STEMI or coronary artery bypass grafting (CABG). **C**, Platelet levels of S100A8 and (**D**) S100A9 were strongly positively correlated with neutrophil count at the time of STEMI, but no correlation was seen at day 3 for either protein.

**Figure 4.**
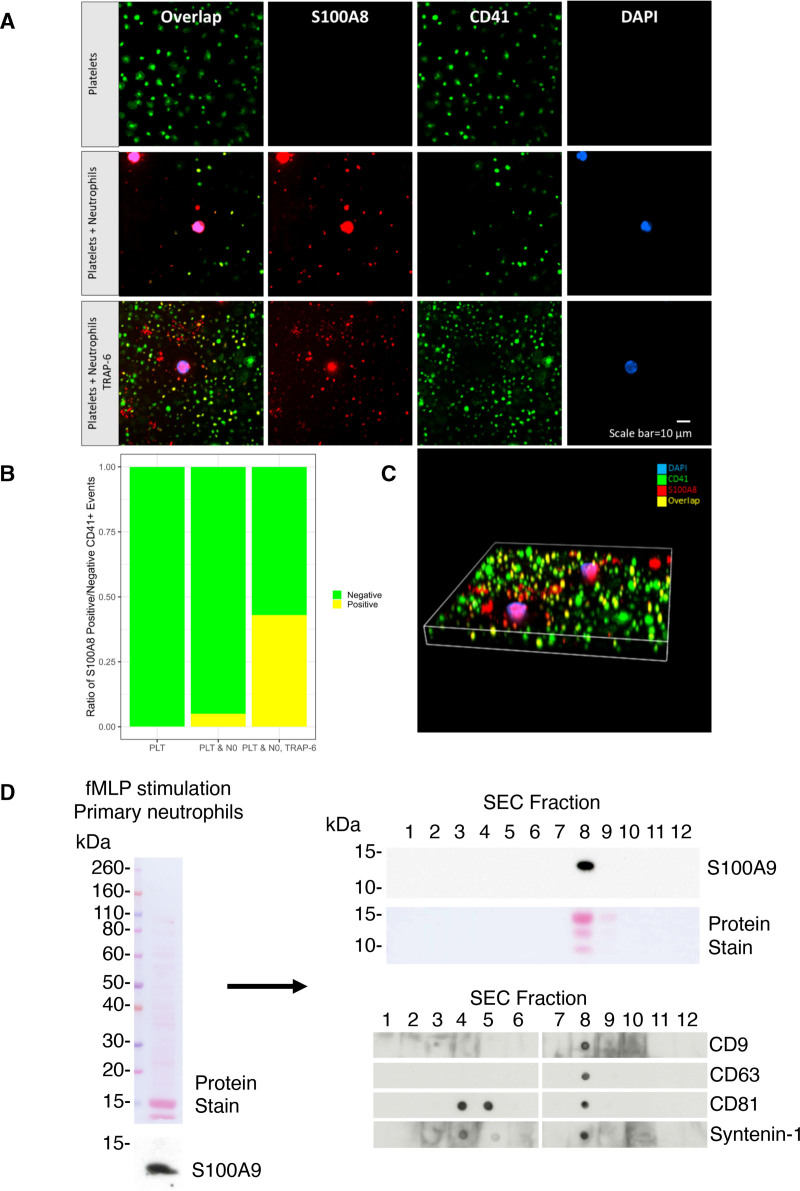
**Platelets internalize protein S100A8 from coactivated neutrophils. A**, To determine the source of platelet S100A8/A9 in patients with STEMI, isolated quiescent platelets (**top**), quiescent platelets coincubated with neutrophils without (**middle**) and with activation by 30-nM TRAP-6 (thrombin-related activating peptide-6; **bottom**) were incubated for 2 h before fixation and staining. Blue color: 4′,6-diamidino-2-phenylindole (DAPI) stain; green color: CD41 (cluster of differentiation); red color: S100A8. Quiescent platelets harbor no S100A8. Staining for S100A8 is primarily observed in neutrophils but also in smaller released neutrophil microparticles. Activation of platelets with TRAP-6 leads to greater release of S100A8 from neutrophils and increased S100A8/CD41 costaining. **B**, Quantification of costaining: 0.01% of 1247 events in quiescent platelets, 5% of 6925 events with quiescent platelets and neutrophils, and 43% of 23 053 events in activated platelets with neutrophils. Event numbers represent 3 replicate fields of view per condition. **C**, Three-dimensional imaging confirms the internalization of S100A8 into platelets when interacting with neutrophils under activating conditions. **D**, Neutrophils were stimulated with 1 µmol/L N-formyl-methionyl-leucyl-phenylalanine (fMLP) for 30 minutes, and the protein releasate was analyzed by Western blot. fMLP-stimulated neutrophil releasate was separated by size-exclusion chromatography (SEC), and S100A9 abundance was determined by Western blot. Vesicle marker elution profiles were determined by dot blot.

We sought to determine the source of platelet S100A8 and S100A9, in the absence of detectable de novo synthesis. As S100A8/A9 has been previously described primarily as a neutrophil-derived protein, we assessed plasma concentration of this heterodimer with ELISAs and compared its abundance with neutrophil count. In CABG patients, plasma S100A8/A9 levels were strongly correlated with neutrophil count (*R*=0.64, *P*=6.5×10^−^^7^; n=25 at 2 time points), whereas in STEMI, this correlation remained but was attenuated (*R*=0.38, *P*=0.0028; n=30 at 2 time points), suggesting, as expected, that neutrophils contribute to plasma levels of S100A8/A9 in both disease conditions (Figure 3A). However, when analyzed per time point, S100A8/A9 was not associated with neutrophil count before or 3 days after CABG (day 0: *R*=0.36; *P*=0.079; FDR, 0.16; day 3: *R*=0.34; *P*=0.1; FDR, 0.16) but was associated at days 0 and 3 in STEMI samples (day 0: *R*=0.45; *P*=0.013; FDR, 0.039; day 3: *R*=0.7; *P*=2×10^−5^; FDR, 8×10^−5^; n=30; Figure 3B). Thus, as expected, neutrophil count is associated with plasma S100A8/A9 levels in patients experiencing STEMI. By contrast, platelet count was not correlated with plasma S100A8/A9 measurements in STEMI (*R*=−0.038; *P*=0.77; FDR, 0.77) and was negatively associated in CABG (*R*=−0.55; *P*=4×10^−5^; FDR, 8×10^−5^). Measurements of S100A8 or A9 in platelets by TMT LC-MS/MS also did not reflect plasma levels of S100A8/A9 heterodimer in patients with STEMI (S100A8: *R*=0.38; *P*=−0.04; FDR, 0.08; S100A9: *R*=−0.048; *P*=0.8; FDR, 0.8), suggesting that S100A8/A9 release from, or plasma contamination of, platelets is insufficient to explain these changes. We then compared platelet S100A8 and S100A9 with neutrophil indices. In STEMI, values correlated strongly at the time of STEMI (day 0)—a relationship which disappeared at day 3 (day 0: S100A8: *R*=0.51; *P*=0.0043; FDR, 0.013; S100A9: *R*=0.54; *P*=0.0025; FDR, 0.010; day 3: S100A8: *R*=0.058; *P*=0.76; FDR, 1; S100A9: *R*=0.031; *P*=0.87; FDR, 1; Figure 3C and 3D). Therefore, we hypothesized that platelet S100A8 and S100A9 abundance is dependent on neutrophils at the time of STEMI. To investigate the potential for direct transfer and uptake of S100A8 from neutrophils to platelets, washed platelets and neutrophils from a healthy volunteer were separated and isolated from whole blood, recombined, and coactivated with TRAP-6. Immunofluorescence analysis demonstrated no detectable S100A8 in quiescent platelets and confirmed its presence in quiescent neutrophils. As seen in Figure 4A through 4C, on coactivation, S100A8 was significantly increased in platelets. *Z* axis reconstruction confirmed the presence of S100A8 protein within, rather than on the surface of, platelets. We, therefore, investigated the mechanism of release of S100A8/A9 from neutrophils. Separation of a neutrophil releasate by size-exclusion LC and subsequent immunoassays demonstrated the presence of S100A8/A9 in neutrophil releasate (Figure 4D). Markers for microvesicles (CD81 and syntenin-1) eluted in the expected early fractions (4 and 5), where S100A8/A9 was not detected, confirming that this protein is not in intact vesicles. However, S100A8/A9 was detected in fraction 8, confirming that S100A8/A9 is not contained in neutrophil microvesicles but released as free heterodimer.

### Neutrophil Activation and Platelet Reactivity

**Figure 5.**
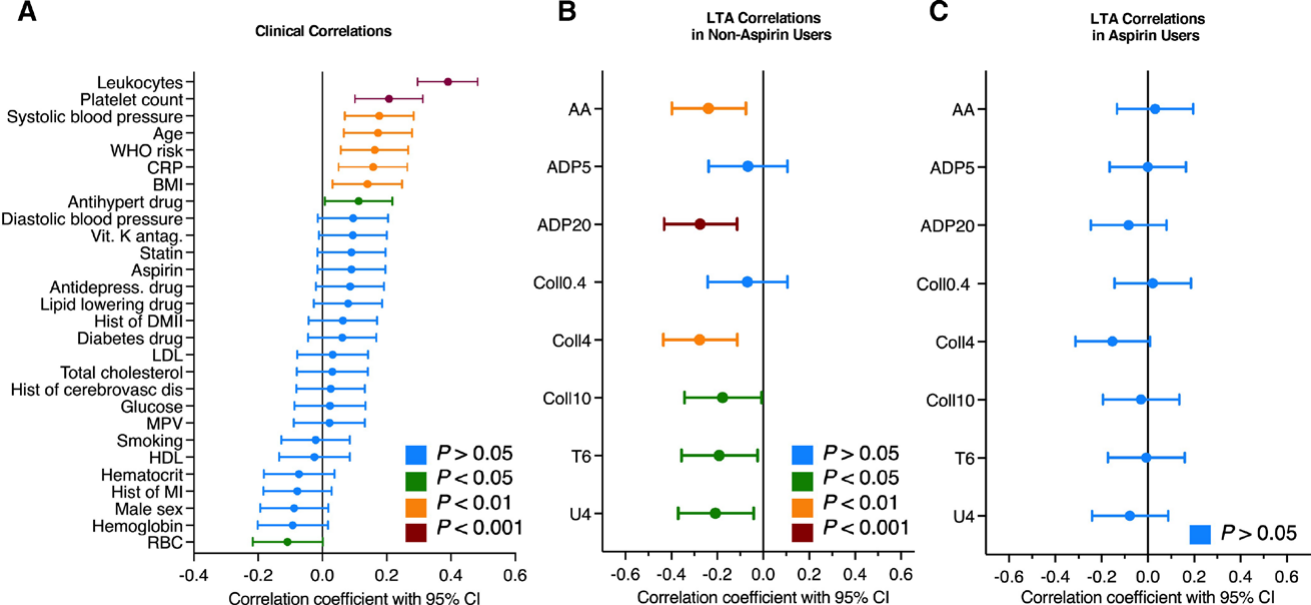
**Correlation of S100A8/A9 in platelet-poor plasma with clinical variables and light transmission aggregometry measurements in a community-based cohort study. A**, Correlation of S100A8/A9 levels in platelet-poor plasma with clinical variables (n=338). **B** and **C**, Correlation of S100A8/A9 levels in platelet-poor plasma with light transmission aggregometry (LTA) measurements in nonaspirin users (**B**; n=185) and aspirin users (**C**; n=153).

To assess the relationship between plasma S100A8/A9 and platelet reactivity, PPP from 338 individuals in the 2015 Bruneck cohort was analyzed using an ELISA and levels compared with clinical data and platelet reactivity measurements using LTA in the same cohort. The baseline characteristics have been described previously.^26,28^ As expected, PPP levels of S100A8/A9 correlated strongly with leukocyte counts (*R*=0.39, *P*<0.00001) and also with platelet counts (*R*=0.21, *P*=0.0001; Figure 5A). S100A8/A9 levels in PPP were negatively correlated with platelet reactivity in nonaspirin users as assessed by LTA with a variety of agonists (1 mmol/L per L arachidonic acid: *R*=−0.24, *P*=0.0039; 20 µM/L ADP: *R*=−0.26, *P*=0.0005; 4 µg/mL collagen: *R*=−0.26, *P*=0.0006; 10 µg/mL collagen: *R*=−0.18, *P*=0.0159; 25 µM/L TRAP-6: *R*=−0.18, *P*=0.0161; 10 µM/L U46619: *R*=−0.20, *P*=0.0079; Figure 5B). This effect was absent for individuals taking aspirin (Figure 5C). Thus, neutrophil-derived S100A8/A9 is associated with reduced ex vivo platelet reactivity in the absence of aspirin.

## Discussion

In this study, we characterized the platelet proteome during acute MI, showing platelet enrichment with S100A8 and S100A9. We excluded cellular contamination of platelets and de novo protein synthesis in platelets as significant contributions to this finding. Instead, we demonstrate transfer of S100A8/A9 from neutrophils to platelets and show that S100A8/A9 is released from neutrophils as free protein. We show that increased plasma S100A8/A9 levels are associated with reduced ex vivo platelet activation to clinically important platelet agonists in a large cohort study. Neutrophil-platelet interactions may impact platelet reactivity measurements.

### S100A8/A9 in the Platelet Proteome During STEMI

In line with previous studies, we found the platelet proteome to be relatively stable at the time of STEMI.^20,21^ In the only other comparable studies, 2-dimensional difference in-gel electrophoresis techniques found <50 differentially regulated proteins in STEMI, without correcting for multiple testing.^20,21^ This is the first study demonstrating increased protein levels of S100A8 and S100A9 in platelets during STEMI, doubling in abundance when comparing the time of STEMI with stable CAD. S100A8 and S100A9 form a heterodimer (S100A8/A9, also known as calprotectin), which is a ligand for TLR4 (toll-like receptor-4), RAGE (receptor for advanced glycation end-products), and CD36. S100A8/A9 is primarily stored in and released from neutrophils on activation. Other potential secondary sources in the circulation are macrophages, monocytes, endothelial cells, and platelets,^29^ but the presence of subthreshold detection of increased LTF and neutrophil DEFA further supports neutrophils as the main source. Increased platelet levels of S100A8/A9 heterodimer have previously been linked with incident MI in patients with systemic lupus erythematosus,^30^ and we have demonstrated its utility as a biomarker in prediction of future cardiovascular events.^31^ High levels of S100A8/A9 are measured in the circulation after MI, as well as other inflammatory illnesses.^32^ Alongside protein measurements, we found platelet mRNA levels for *S100A8* and *S100A9* were increased at the time of STEMI when compared with platelets 3 days later. This change in *S100A8* and *S100A9* levels was not seen after CABG, our comparator thromboinflammatory environment. Platelet levels of *S100A9* mRNA have previously been elevated at the time, and associated with the risk, of MI.^33^

### Lack of Protein Synthesis in Platelets?

In contrast to the current paradigm, we were unable to demonstrate de novo protein synthesis in platelets using SILAC proteomics, despite detecting low levels of synthesis in leukocytes. Platelets have been shown to synthesize new protein, primarily on activation, and contain the relevant translational machinery.^34–42^ Almost all the preexisting data on de novo protein synthesis depend on the incorporation of ^35^methionine into protein—a method far more sensitive than LC-MS/MS. Further, a small proportion of platelets are reticulated—the population thought to harbor the greatest synthetic potential.^34^ It is, therefore, possible that the synthesis of protein in platelets is below the threshold of detection for LC-MS/MS, although the doubling of S100A8 and S100A9 abundance at the time of MI, and the absence of evidence for their synthesis in platelets by any method of detection, means synthesis in platelets is unlikely to explain our findings. Functional relevance of low levels of platelet protein synthesis is challenged by the fact that platelets lose around half of their total protein content over the course of their circulating life.^43^ An alternative source of *S100A8* and *S100A9* are megakaryocytes. While recent single-cell transcriptomic analysis of primary human megakaryocytes found *S100A8* and *S100A9* to be differentially expressed in 32 N compared with 4 N megakaryocytes, no increase in these transcripts was seen in megakaryocytes harvested at the time of MI.^44^ By contrast, however, S100A8/A9 has been shown to be directly released from neutrophils recruited to the necrotic myocardium in acute MI.^45,46^ The transfer of S100A8 to platelets offers an alternative mechanism for protein enrichment.^47^

### S100A8 Is Released From Activated Neutrophils and Internalized by Platelets

We sought an ectopic source of S100A8/A9 protein in platelets. Plasma S100A8/A9 in patients was strongly associated with neutrophil count in the CABG group (*R*=0.66, *P*=2.3×10^−7^) and moderately associated with neutrophil count in the STEMI group (*R*=0.28, *P*=0.032). These data agree with the strong relationship between neutrophil count and plasma S100A8/A9 seen in healthy individuals, those with lupus, those with cardiovascular disease risk factors, and in mechanistic studies in mice but extend the findings beyond plasma and into the platelet.^29,30,48^ When a platelet-neutrophil admixture was activated with TRAP-6, around 30% of platelets demonstrated uptake of S100A8. While RNA analysis rules out differential cellular contamination, it remains possible that the isolated platelet fraction in STEMI samples contained neutrophil extracellular vesicles. Also, other products of neutrophil behavior, namely neutrophil extracellular traps, the recently described elongated neutrophil-derived structures, or neutrophil-megakaryocyte emperipolesis, could account for these changes.^49–54^ In activated neutrophils, however, we demonstrate that S100A8/A9 is released as a free protein heterodimer. This replicates the findings of a similar proteomic analysis of neutrophil releasate^55^ and supports previous findings that blocking of CD36 prevents uptake of S100A8/A9 in platelets forming in whole blood.^56^

### S100A8/A9 and Platelet Reactivity

In the largest published cohort to undergo LTA to date, we found that plasma S100A8/A9 was correlated with leukocyte count and was correlated with platelet count in aspirin users, supporting a primary neutrophil source in plasma, as well as the previously described link between S100A8/A9 and thrombopoiesis.^48^ S100A8/A9 plasma levels were negatively associated with platelet reactivity. The direction of this effect is robust but unexpected: antibody inhibition and genetic knockout experiments in small animals have indicated a prothrombotic association for S100A8/A9.^56–58^ Thrombosis assays are heterogenous, for example, S100A9^−^^/^^−^ mice show reduced thrombus formation and relative P-selectin expression in response to collagen exposure in whole blood but do not demonstrate prolonged tail bleeding times.^56^ However, the prevailing direction of previous experimental evidence in animals supports the hypothesis that S100A8/A9 promotes, rather than prevents, thrombosis. The most likely explanation for the unexpected inverse association with platelet activation is that elevated plasma levels of S100A8/A9 reflect neutrophil activation, leading to the preactivation of platelets in vivo and platelet exhaustion when instantaneous platelet activation is then assessed ex vivo. Accordingly, when platelet activation in patients with stable and unstable CAD has been assessed by urinary 11-dehydro-thromboxane β2 levels, which may reflect platelet activity over a longer period of time, a positive correlation with plasma S100A8/A9 was shown.^59^ Aspirin therapy may prevent platelet preactivation by neutrophils. Thus, we only observed an inverse correlation between S100A8/A9 plasma levels and LTA in nonaspirin users but not aspirin users. The effect of in vivo neutrophil-platelet interactions may explain, in part, the challenges faced when using LTA to guide antiplatelet therapy at the time of MI. This raises the question of whether other platelet function markers, which may reflect in vivo reactivity, such as circulating microRNAs whose levels integrate platelet activation over time, may be better suited to capture these processes, as we have discussed previously.^60^

### Inflammation in Acute Coronary Disease: Trigger or Response?

Protein S100A8/A9, along with LTF and DEFA1 to a lesser degree, constitutes neutrophil-derived alterations to the platelet proteome during MI. Unsurprisingly, elevations of platelet and plasma S100A8/A9 and neutrophil count were also seen in the positive control group: patients 3 days after CABG. This confirms the role of innate immune activation, likely in the necrotic myocardium,^45,46^ but also potentially from ruptured plaque,^31,61^ in the enrichment of S100A8/A9 in platelets. Large clinical trials have recently demonstrated reductions in MI using drugs targeting inflammatory axes both as primary^62^ and secondary prevention.^63,64^ The degree to which thrombosis and inflammation overlap, regulate, and potentiate one another is increasingly scrutinized.^65^ Our study adds to this growing body of work by identifying S100A8/A9 as a molecular target for further investigation or therapies, alongside previously identified neutrophil extracellular traps and cell-free histones.^52,66^ Further work is required to determine on whether the inflammatory platelet signature we describe in STEMI, and its subsequent effect on reactivity, can be used to identify those with high risk of plaque rupture.

### Conclusions

While changes in the platelet proteome have been previously described in acute cardiovascular disease, this study demonstrates the transfer of protein into platelets. In the largest and deepest proteomic analysis of platelets in MI to date, we discovered the surprising stability of the platelet proteome and identified alarmins S100A8 and S100A9 as the major alterations. Our sampling strategy, preparing platelet lysate at the time of emergency presentation, offers the closest insights so far into the platelet protein composition at the time of MI. Therefore, the high degree of proteomic stability between platelets during STEMI in comparison with stable CAD does not support the hypothesis that widespread changes in the platelet proteome precede or can be considered to be causal in the acute pathogenesis of MI, especially as the changes detected were reproduced in vitro.^15,16^ Platelet S100A8 and S100A9 abundance was strongly related to neutrophil indices at the time of STEMI, and we demonstrate transfer of S100A8 from neutrophils to platelets within the time frames associated with the pathophysiology of STEMI, likely as free rather than vesicular protein. Plasma levels of neutrophil-derived S100A8/A9 are associated with reduced ex vivo platelet reactivity to endogenous agonists, most probably due to platelet preactivation in vivo, which can be prevented by aspirin. Alterations in platelet proteome during STEMI, therefore, appear to be primarily due to platelet-neutrophil interactions—an axis that may present a future target for prevention of thrombotic complications in CAD and that may, in part, explain previous failures of ex vivo platelet reactivity testing in the setting of MI.

## Article Information

### Acknowledgments

Drs Christian Schulte and Anna Zampetaki advised on mRNA experimental protocols. Dr Kaloyan Takov established the HPLC fractionation protocol. Mervyn Andiapen, Brian Piniera, and Prof Rakesh Uppal (Bart’s Heart Centre) supported clinical recruitment. Elka Giemza and Dr Jonathan Hill (King’s College) supported sampling from healthy participants. All the patients who agreed to participate are gratefully acknowledged. Finally, we would like to thank the Nikon Imaging Centre and Wohl Cellular Imaging Centre at King's College London. A. Joshi conceived the study, recruited the patients, designed and performed the experiments, interpreted the results, and wrote the manuscript. M. Mayr conceived the study, designed the experiments, interpreted the results, and wrote the manuscript. A. Mathur designed the study, recruited the patients, and advised on the manuscript. L.E. Schmidt, S.A. Burnap, T. Barwari, R. Lu, C. Gutmann, M.V. Chan, P.C. Armstrong, and F. Baig performed the experiments and advised on the manuscript. S. Kiechl and T.D. Warner conceived the study. K. Theofilatos and P. Willeit advised on the manuscript.

### Sources of Funding

A. Joshi is a British Heart Foundation (BHF) Clinical Research Training Fellow (FS/16/32/32184). C. Gutmann is funded by a BHF PhD studentship (FS/18/60/34181). M. Chan and P. Armstrong were supported by grants from the British Heart Foundation (PG/15/47/31591 and RG/19/8/34500). A. Mathur receives funding from the National Institute of Health Research, the Small Business Research Initiative, Bart’s Charity, and Abbott Vascular. M. Mayr is a BHF chair holder (CH/16/3/32406) and receives BHF programme grant support (RG/16/14/32397). Research by M. Mayr was made possible through support from the BIRAX Regenerative Medicine Initiative and funding from the EU Horizon 2020 Research and Innovation Programme under Marie Skłodowska-Curie grant agreement No. 813716 (TRAIN-HEART), the Leducq Foundation (13CVD02 and 18CVD02), the Excellence Initiative (Competence Centers for Excellent Technologies) of the Austrian Research Promotion Agency (FFG): “Research Center of Excellence in Vascular Ageing—Tyrol, VASCage” (K-Project No. 868624 and 843536) funded by the Austrian Ministry for Transport, Innovation and Technology, the Austrian Ministry for Digital and Economic Affairs and the federal states Tyrol (via Standortagentur), Salzburg, and Vienna (via Vienna Business Agency), and the National Institute of Health Research Biomedical Research Centre based at the Guy’s and St Thomas’ National Health Service Foundation Trust and King’s College London in partnership with the King’s College Hospital.

### Disclosures

None.

### Supplemental Material

Supplemental Methods

Figures S1 through S4

Tables S1, S3, and S4

## Supplementary Material


