# Supplementary material for: Neutrophil-Derived Protein S100A8/A9 Alters the Platelet Proteome in Acute Myocardial Infarction and Is Associated With Changes in Platelet Reactivity
Source: Arterioscler Thromb Vasc Biol. 2021 Nov 23;42(1):49–62. doi: 10.1161/ATVBAHA.121.317113 (PMC8691374; doi:10.1161/ATVBAHA.121.317113)
Supplement: Supplementary file 1 [file atv-42-049-s001.pdf]

SUPPLEMENTAL MATERIAL

Neutrophil Derived Protein S100A8/A9 Alters the Platelet Proteome in Acute Myocardial Infarction and Is Associated with Changes in Platelet Reactivity

Abhishek Joshi[1,2], Lukas E. Schmidt[1], Sean A. Burnap[1], Ruifang Lu[1], Melissa V Chan[3], Paul C. Armstrong[3], Ferheen Baig[1], Clemens Gutmann[1], Peter Willeit[4], Peter Santer[5], Temo Barwari[1], Konstantinos Theofilatos[1], Stefan Kiechl[4,6,7], Johann Willeit[4,6,7], Tim Warner[3], Anthony Mathur[2,8], Manuel Mayr[1].

1 SUPPLEMENTARY METHODS

1.1 INCLUSION AND EXCLUSION CRITERIA FOR PATIENTS RECRUITED FROM THE BART'S HEART CENTRE

1.1.1 Inclusion and Exclusion Criteria: STEMI

Inclusion criteria were as follows:

1. Aged 18-80
2. Capable of providing informed consent
3. Experiencing STEMI by clinical and ECG criteria (typical chest pain, with ST elevation on ECG >2mm in 2 contiguous chest leads, or >1mm in 2 contiguous limb leads, or new left bundle branch block)
4. Symptom onset less than 6 hours prior to presentation.
5. Considered to require emergency angiography with possible angioplasty
6. Found to have a coronary artery occluded with acute thrombus on angiography
7. Found to have evidence of atherosclerotic disease on angiography

Exclusion criteria:

1. Already taking antiplatelet therapy.
2. Previous diagnosis of MI.
3. Previous diagnosis of heart, renal or liver failure.
4. Previous or subsequent diagnosis of platelet function or production disease (such as idiopathic thrombocytopenia)
5. Experiencing haemorrhage resulting in loss of >1g/dl of haemoglobin within the 3 day study period, or requiring red cell, platelet or plasma transfusion as part of treatment of haemorrhage.

1.1.2 Inclusion and Exclusion Criteria: CABG

Inclusion criteria

1) Aged 18-80
2) Capable of providing informed consent
3) Undergoing elective (not emergency or urgent) cardiopulmonary bypass surgery

Exclusion criteria

1) History of MI within a year of recruitment.

2) Previous diagnosis of heart, renal or liver failure.
3) Previous or subsequent diagnosis of platelet function or production disease (such as idiopathic thrombocytopenia)
4) Requiring platelet transfusion as part of usual clinical care.

1.2 Preparation of Platelet Poor Plasma and Platelet Pellet Samples

Whole blood tubes were centrifuged at 200 x *g* for 15 minutes, with centrifuge acceleration and deceleration set to 0. On completion the supernatant, platelet rich plasma (PRP), was transferred to 2 fresh Falcon tubes and the Buffy and red cell pellet discarded. The volume of PRP was noted and 1mM prostacyclin (PGI_2; Tocris Bioscience, cat. no. 2989) was added to final concentration of 1nM. Prostacyclin was chosen for its short half-life, allowing subsequent activation of platelets and collection of releasate. Falcon tubes were then centrifuged at 1000x*g* for 10 minutes, again without acceleration or brakes. The resulting supernatant, platelet poor plasma (PPP) was aspirated, and two 2ml aliquots transferred to 1.5ml Eppendorf tubes and a single 250µl aliquot transferred to a 450µl glass cuvette (Labmedics, Catalogue No. 300-0312) for preparation of platelet releasate. The remaining platelet pellet was resuspended in 5ml of modified Tyrodes-HEPES solution without bovine serum albumin, (NaCl, 134 mM; KCl, 2.9 mM; Na_2HPO_4, 0.34 mM; $NaHCO_3$, 12 mM; HEPES, 20 mM; $MgCl_2$, 1 mM) in each Falcon tube, again supplemented to 1nM PGI_2, and centrifuged again at 1000x*g,* without acceleration or brakes as a first washing step. The supernatant was discarded and the resultant pellets resuspended in 4mls of Tyrodes, 250µl removed to a glass cuvette, and again supplemented to 1nM PGI_2 and then split into 4 separate 1ml aliquots in 1.5ml Eppendorf tubes. These were again centrifuged at 1000x*g* without brakes or acceleration. The supernatant was again removed, leaving 4 individual platelet pellets. One pellet was resuspended and immediately lysed in 500µl lysis buffer cocktail supplemented by protease inhibitor and phosphatase inhibitor (329µl milliQ water, 50µl of 10X Cell Lysis Buffer [Cell Signalling Technology, Catalogue Number #9803], 71µl of 7X protease inhibitor cocktail [Merck, catalogue number 11697498001], and 50µl of phosphatase inhibitor [PhosSTOP, Merck, catalogue number: 4906845001]). A second pellet was lysed in 500µl Qiazol (Qiagen, Catalogue No. 79306). All samples and were frozen immediately at -80°C within 2 hours of blood draw.

1.3 MASS SPECTROMETRY ANALYSIS OF PLATELET PELLET IN A CLINICAL COHORT

1.3.1 **In-solution Digestion of Platelet Lysate**

Samples were thawed, sonicated twice, centrifuged at 14,000x*g* at 4°C, and protein concentrations determined via BCA Assay. 50µg of protein from each sample was transferred to a fresh 2.0ml Eppendorf and volumes equalised to 80µl with deionised water. Samples were then denatured by addition of 160µl of 9M/3M Urea/Thiourea to a final concentration of 6M/3M, and reduced through addition of 26.7µl of 100mM Dithiothreitol (DTT) to a final concentration of 10mM, and incubated for 1 hour at 37°C. Samples were alkylated through the addition of 29.6µl of 500mM Iodoacetamide (IAA) to a final concentration of 50mM, and incubated for 1hr in the dark. Samples were then precipitated through the addition of excess acetone (ratio 6:1) overnight at -20°C, centrifuged at 16,000x*g* for 40 minutes to pellet the precipitated protein and them supernatant removed leaving approximately 50µl, then dried down using a SpeedVac and resuspended in 90µl of 0.1M triethylammonium bicarbonate (TEAB) and placed on a shaker for 30 minutes to fully resuspend the protein pellet. Reduced, alkylated protein in 0.1M TEAB was then digested with Trypsin/LysC (Promega, Catalogue Number V5072) at a ratio of 1:50 with protein (10µl of 0.1µg/ul of Trypsin/LysC in 50mM acetic acid was added to each sample), and incubated for 18 hours at 37°C.

1.3.2 **TMT 11-Plex Tagging of Platelet Lysate**

Digestion was stopped by placing samples on ice. 10µg (20µl) of each sample was transferred to a fresh 1.5ml Eppendorf tubes. 3µl of each sample was combined in a further fresh Eppendorf and constituted the sample pool. 256µl of acetonitrile was added to each of 11 TMT-tags. Groups were designated as below:

TMT TAG	Group 1	Group 2	Group 3	Group 4	Group 5	Group 6	Group 7	Group 8	Group 9	Group 10	Group 11		
126	S1	C4.1	C7.1	C9	C11	S18.1	S21.1	S24.1	S28	S30	C25.1		STEMI Day 0 Samples
127N	S2	S5	C8.1	C9.1	C12	C13	S22.1	S26.1	S28.1	S31	C26.1		STEMI Day 3 Samples
127C	S4	S6	S8	C10.1	C11.1	C14	C15	S27.1	S29.1	S30.1	C27.1		CABG Day 0 Samples
128N	S1.1	S7	S9	S11	C12.1	C13.1	C16	C17	C19	S31.1	S32.1		CABG Day 3 Samples
128C	S2.1	S5.1	S10	S12	S15	C14.1	C15.1	C18	C20	C22	S33.1		
129N	S4.1	S6.1	S8.1	S14	S16	S18	C16.1	C17.1	C21	C23	C25		
9C	C2	S7.1	S9.1	S11.1	S17	S19	S21	C18.1	C19.1	C24	C26		
130N	C3	C4	S10.1	S12.1	S15.1	S20	S22	S24	C20.1	C22.1	C27		
130C	C2.1	C5	C7	S14.1	S16.1	S19.1	S23	S26	C21.1	C23.1	S32		
131N	C3.1	C5.1	C8	C10	S17.1	S20.1	S23.1	S27	S29	C24.1	S33		
131C	POOL	POOL	POOL	POOL	POOL	POOL	POOL	POOL	POOL	POOL	POOL		

Table SI: *Arrangement of TMT groups in the clinical cohort of platelet pellet lysate*

Each TMT group contained samples from both CABG and STEMI groups, and from both day 0 and day 3 samples, and where each participant's samples were analysed, as far as possible, within the same group. 8.2ul of the relevant tag (ratio 0.41:1) was added to each sample, and 135µl of tag added to the pool. Samples were incubated at room temperature for 1 hour and then quenched with the addition of 1.7µl of 5% hydroxylamine to each sample, and 27.9µl to the pool. 29.9µl of each relevant tagged sample and 30µl of tagged pool were then combined in a single fresh Eppendorf to create 11 Groups (**Table SI**), ready for high pH reversed phase peptide fractionation.

1.3.3 High pH Reverse Phase Peptide Fractionation of TMT-Tagged Platelet Lysate Peptide

Eleven fractionation columns (Pierce High pH Reversed-Phase Peptide Fractionation Kit, ThermoScientific catalogue 84868) were prepared for use, first through centrifugation at 5000 x *g* for 2 minutes to pack the resin, and then with the addition of 300µl acetonitrile and further centrifugation at 5000 x *g*. This wash step was performed twice. Columns were then washed once with 300ul of 0.1% Trifluoroacetic acid (TFA) solution.

TMT groups were dissolved in 300µl of 0.1% TFA, and loaded onto the fractionation column in a fresh Eppendorf and centrifuged at 3000x*g* for 2 minutes. Flow-through was collected and the columns transferred to a fresh Eppendorf, loaded with 300µl of deionised water and centrifuged at 3000x*g* for 2 minutes. Flow-through was stored, labelled as "wash", then washed again with 300µl of 5% acetonitrile with 0.1% triethylamine. Each column was then loaded with 300µl of fraction solutions of increasing ACN concentration (see **Table S2**), centrifuged at 3000x*g* for 2 minutes and the fractions collected.

FRACTION	ACN [%]	ACN [µl]	0.1 % TEA [µl]
**TMT wash**	5.0	200	3800
**1**	10.0	400	3600
**2**	12.5	500	3500
**3**	15.0	600	3400
**4**	17.5	700	3300
**5**	20.0	800	3200
**6**	22.5	900	3100
**7**	25.0	1000	3000

**8**	50.0	2000	2000

Table SII: *Composition of fractionation solvents for TMT-tagged peptides, by reducing hydrophobicity. ACN = acetonitrile, TEA = triethylamine*

After fractionation, eight fractions per grouped TMT sample were vacuum-centrifuged to dryness and reconstituted in 83 µl of 2 % (v/v) acetonitrile and 0.05 % (v/v) TFA. Samples were then transferred to a mass spectrometry plate, ready for injection.

1.3.4 **Liquid chromatography- tandem mass spectrometry (LC-MS/MS)**

TMT-labelled, fractionated peptides were analysed using an LC-MS/MS assembly consisting of a nano-flow UltiMate 3000 high-performance liquid chromatography (HPLC) system (Thermo Scientific) coupled via an EASY-Spray Source (Thermo Scientific) to an Orbitrap Fusion Lumos Tribrid mass spectrometer (Thermo Scientific).

Peptides were trapped on a C18 cartridge (5 mm x 0.3 mm, particle size 5 µm, pore size 100 Å, Thermo Scientific) and separated using an EASY-Spray C18 column (75 µm x 50 cm, particle size 2 µm, pore size 100 Å, Thermo Scientific), which was kept at 45°C. For each LC-MS^3 analysis, 10 µl of reconstituted peptide fraction were loaded onto the trap cartridge at a flow rate of 25 µl/min for 3 min, and eluted from it at a flow rate of 0.25 µl/min using the following gradient: 0–10 min, 4–10 % B; 10–75 min, 10–30 % B; 75–80 min, 30–40 % B; 80–85 min, 40–99 % B; 85–89.8 min, 99 % B; 89.8–90 min, 99–4 % B; 90–120 min, 4 % B; with A being 0.1 % (v/v) formic acid and B being 80 % (v/v) acetonitrile + 0.1 % (v/v) formic acid.

MS data was acquired using a Synchronous Precursor Selection (SPS)-MS^3 method with a cycle time of 3s. An Orbitrap full MS scan (scan range 375–1500 m/z; resolution 120,000; automatic gain control target $4e^5$; max. injection time 50ms) was followed by an ion trap data-dependent MS^2 scan using collision-induced dissociation (CID) fragmentation (dynamic exclusion duration 60s, MS isolation window 0.7 m/z; CID collision energy 35 %; automatic gain control target 1e4; max. injection time 50ms), and an Orbitrap data-dependent SPS-MS^3 scan using higher-energy C-trap dissociation (HCD) fragmentation to generate TMT reporter ions from 5 SPS precursors (scan range 100-500 m/z; resolution 60,000; MS^2 isolation window 2 m/z; HCD collision energy 65 %; automatic gain control target 1e5; max. injection time 105ms).

1.3.5 **Database search**

Proteome Discoverer software (version 2.2.0.388, Thermo Scientific) was used to search raw data files against the human database (version May 2018, UniProtKB/Swiss-Prot) using Mascot (version 2.6.0, Matrix Science). The mass tolerance was set at 10 ppm for precursor ions and 0.8 Da for fragment ions. Trypsin was set as the protein-digesting enzyme with up to two missed cleavages being allowed. Carbamidomethylation of cysteine was set as a static modification, and oxidation of methionine was set as a dynamic modification. To detect TMT-labelled peptides, the modifications specified in the quantification method (TMT-11plex, lysine and N-terminal residue modification +229.163 Da) were included in the search. Correction for the reagent lot-specific isotopic impurities of TMT tags was applied (TMT-10plex lot no. TD266157; TMT11-131C lot no. TD264064). To account for variation in abundances between samples, data was normalised to the total peptide amount. After normalisation, protein abundance was scaled to the average abundance of the pooled sample.

1.4 RNA ANALYSIS OF PLATELETS

1.4.1 **RNA extraction**

RNA was extracted from platelets, plasma or platelet releasate using the QIAGen miRNAEasy mini kit (Cat. No. 217004), following manufacturer's instructions. Sample pellets were lysed in 500µL of Qiazol at the time of harvest. 100µl of thawed, centrifuged plasma or platelet releasate was added to 500µL of Qiazol. A mixture of synthetic cel-miR-39-3p and carrier RNA (MS2 RNA, Roche) was added in a volume of 200 µl QIAzol reagent. Following brief mixing and incubation at room temperature for 5 minutes, 140 µl of chloroform (Fisher Scientific, Cat. No. 12832224) was added and the solution was mixed vigorously for 20 seconds, and further incubated at room temperature for 5 minutes. Samples were then centrifuged at 13,500 x *g* for 15 min at 4°C. 280 µl of upper (aqueous) phase was carefully mixed with 420 µl of 100% ethanol and then applied to columns and washed three times according to the manufacturer's protocol. First, 700µl of RWT buffer was added, the column centrifuged at 12,000 x *g* for 1 minute, and the flowthrough discarded. Then, 500µl RPE buffer was added and the column centrifuged at 13000 x *g* for 1 minute, and the flowthrough again discarded. 500µl RPE buffer was again added, and the column centrifuged at 13000 x *g* for 2 minutes. Flowthrough was again discarded. Columns were transferred to a dry collection tube and centrifuged at 15000 x *g* for 1 minute to dry. Finally, RNA was eluted by the addition of

35 µl of nuclease-free H_2O and centrifugation at 9000 x *g* for 1 min at room temperature into a fresh Eppendorf. RNA samples were stored at -80°C prior to further analysis.

1.4.2 **Heparinase Treatment of RNA**

Previous assessments of RNA in samples from patients with acute cardiovascular disease have demonstrated significant interference by systemic heparin. Whilst Day 0 samples were collected before the administration of heparin, and day 3 samples collected a significant period afterwards. Therefore, a representative subset of samples were assessed in parallel with and without treatment with heparinase, to determine the effects on this cohort. RNA from 22 PPP samples, 22 releasate samples and 44 platelet samples, containing equal numbers of paired timepoints and treatment groups were selected. 7µl of RNA was combined with 7µl of a heparinase working solution of heparinase (1mg/ml), Ribolock and heparinase buffer (200mM Tris, 40mM $CaCl_2$, 500mM NaCl adjusted to a pH of 7.5), incubated for 3 hours at 25°C. Samples were analysed using qPCR to measure spiked-in *cel-39-3p* as described in subsequent sections. As no difference in the variability of the treated and untreated samples were seen (n = 118, S.D. untreated = 0.554, treated = 0.68), and the raw Cq values lower in the treated group, the remaining clinical cohort samples were measured without heparinase treatment.

1.5 Isolation of Samples for SILAC-labelling Analysis of Protein Synthesis

1.5.1 **Isolation of Platelets and Incubation in SILAC medium**

For 6-hour incubations, whole blood was samples from 5 healthy male individuals, and PRP generated as previously described. Samples were incubated for 6 hours in line with data suggesting the synthetic capacity of reticulated platelets is short-lived. PRP was centrifuged at 1000 x *g* for 10 minutes in a 15ml Falcon tube to generate a platelet pellet, and then washed once with modified Tyrode's-HEPES. Samples from each individual were resuspended in 3ml of adapted phenol-free RPMI cell culture medium (ThermoFisher, catalogue number: A2494201) supplemented with $^{13}C_6$-labelled heavy isotope lysine (ThermoFisher; cat no: 89988) and arginine (ThermoFisher; cat no: 88210) to a final concentration of 0.274mM $^{13}C_6$-Lysine and 1.15mM $^{13}C_6$-Arginine. Resuspended pellet from each individual was then aliquoted into 3 separate 1.5ml Eppendorf tubes. For each individual, one aliquot was incubated

at 37°C for 1 hour to allow equilibration in medium, then centrifuged at 1000x*g* to pellet the platelets, the supernatant separated from the pellet and stored, and the pellet lysed in a cell lysis cocktail consisting of 1x cell lysis buffer (Cell Signalling Technology, catalogue number: 9803S) supplemented with protease inhibitor. A second aliquot was incubated for 6 hours in a heated shaker at 180rpm, at 37°C. A third aliquot was activated with 10µl of 1mM TRAP-6 and incubated for 6 hours in a heated shaker at 180rpm, at 37°C. After incubation, these samples were centrifuged at 1000x*g*, the supernatants removed and stored, and the pellets again lysed in cell lysis cocktail.

The protocol was repeated with 3 technical replicates from 2 healthy individuals, but with 24 hours of incubation, to confirm the findings from the initial 6 hour incubation.

1.5.2 **Isolation of Leukocytes and Incubation in SILAC Medium**

To determine the contribution of white cells to the pool of newly synthesised protein, buffy coat was isolated from 2 healthy individuals. Blood was sampled from the antecubital fossa into 2, 8ml EDTA tubes per individual. Whole blood was then centrifuged at 1000 x *g*. Supernatant plasma was removed leaving a thin layer above the visualised Buffy coat. The buffy coat was then carefully aspirated using a Pasteur pipette and transferred to a fresh Eppendorf. Light microscopy confirmed the presence of mononuclear and polymorphonuclear cells. Cells were washed once in 5ml Hanks' Balanced Saline Solution (HBSS, Sigma-Aldrich, catalogue number H6648) to remove plasma-derived amino-acids. Cell counts were performed using a haemocytometer, pelleted again and resuspended in RPMI containing heavy labelled lysine and arginine, and adjusted to 500,000 cells/ml. $5x10^5$ cells were then plated in a 12-well plate and incubated for 24hours at 37°C in 5% CO_2. After 24 hours cells were adherent to the well bottom, supernatant was removed from the wells and 500µl of cell lysis buffer supplemented with protease inhibitor was added directly to the cells, lysing the cells *in situ*. Lysed sample was transferred to fresh 1.5ml Eppendorf tubes and stored at -80°C.

1.5.3 **Culture of MEG-01 Cells and Incubation in SILAC Medium**

Cells from a megakaryoblastic leukaemia cell line (MEG-01, ATCC) were cultured in RPMI-1640 medium (Life Technologies, cat. no. 52400) supplemented with 10% fetal bovine serum, 100U/ml penicillin and 100 µg/ml streptomycin at 37°C in a humidified atmosphere of 5% CO_2 for 2 passage generations, until sufficient cells were available. Cells were then washed twice in lysine and arginine-free RPMI, counted with haemocytometer, and two replicates of 500,000

cells were resuspended in SILAC amino acid supplemented RPMI medium as previously described and incubated for 24 hours serum-free at 37°C in 5% CO_2. After incubation, adherent cells were loosened from the well with a cell scraper, all cells transferred to 1.5ml Eppendorfs and centrifuged at 1000x*g* to form a pellet. The supernatant was removed, and the pellet lysed in 500µl cell lysis buffer supplemented with protease inhibitor.

1.5.4 **Mass Spectrometry Analysis of SILAC-Incubated Samples**

1.5.4.1 In-Solution Digestion

Protein concentration for platelet pellet was confirmed using BCA assay. 30µg of sample was used and volumes equalised. Samples then underwent in-solution digestion with a 1:20 trypsin ratio.

1.5.4.2 C18 Clean-Up of SILAC Labelled Samples

Peptide samples were purified using a 96-well C18 spin plate (MicroSpin, Harvard Apparatus, cat. no. 745617). The resin was activated using 200µl methanol and centrifuged at 1000 x g for 1 minute. Resin was then cleaned with 200µl of 80% acetonitrile, 0.1% trifluoroacetic acid in H_2O, and three washing steps using 200µl of 1% acetonitrile, 0.1% trifluoroacetic acid in H_2O with centrifugation (1000 x g for 1 minute) after each step. Samples were loaded onto the resin and centrifuged at 2250 x g for 1 minute; the flow through was reloaded onto the resin a second time and centrifugation repeated. The resin was then washed three times with 200 µl1% acetonitrile, 0.1% trifluoroacetic acid in H_2O (centrifugation at 2250 x g for 1 minute). Finally, the samples were eluted with 170µl of 50% acetonitrile, 0.1% trifluoroacetic acid in H_2O (centrifugation at 1000 x g for 1 minute); this step was repeated, combining the collected eluate. Samples were then dried down using the SpeedVac Concentrator for approximately 5 hours. Dried peptide samples were then resuspended in 0.05% trifluoroacetic acid in 2% acetonitrile.

1.5.4.3 Liquid chromatography-mass spectrometry and SILAC Search strategy

Peptides were analysed using an LC-MS assembly consisting of a nano-flow UltiMate 3000 high-performance liquid chromatography system (Thermo Scientific) coupled via an EASY-Spray Source (Thermo Scientific) to a Q Exactive HF mass spectrometer (Thermo Scientific), which was configured for data-dependent acquisition using a full MS/data-dependent MS2 setup.

Peptides were trapped on a C18 cartridge (5 mm x 0.3 mm, particle size 5 μm, pore size 100 Å, Thermo Scientific) and separated using an EASY-Spray C18 column (75 μm x 50 cm, particle size 2 μm, pore size 100 Å, Thermo Scientific), which was kept at 45 °C. Per LC-MS2 analysis, 1 μg of reconstituted peptide was loaded onto the trap cartridge at a flow rate of 25 μl/min for 3 min using 0.1% formic acid (FA) in H2O, and eluted from it at a flow rate of 0.25 μl/min using the following gradient: 0–5min, 4–10% B; 5–75min, 10–30% B; 75−80min, 30−40% B; 80–85min, 40–99% B; 85–89.8min, 99% B; 89.8–90min, 99–4% B; 90–120min, 4% B; with A being 0.1 % (v/v) FA and B being 80 % (v/v) acetonitrile + 0.1 % (v/v) formic acid.

Survey full scan spectra were acquired over the mass-to-charge (m/z) range 350–1600 (resolution 60,000; automatic gain control target 1e6; max. injection time 100 ms). Data-dependent MS2 scan was performed using the top 15 ions in each full scan (resolution 15,000; automatic gain control target 5e4; max. injection time 100 ms; MS isolation window 2.0 m/z; dynamic exclusion duration 40 s).Proteome Discoverer software (version 2.2.0.388, Thermo Scientific) was used to search raw data files against the human database (version Jan 2019, UniProtKB/Swiss-Prot) using Mascot (version 2.6.0, Matrix Science). The mass tolerance was set at 10 ppm for precursor ions and 20 mmu for fragment ions. Trypsin was set as the protein-digesting enzyme with up to two missed cleavages being allowed. Carbamidomethylation of cysteine was set as a static modification, and oxidation of methionine was set as a dynamic modification. To detect stable isotope-labelled peptides, the modifications specified in the quantification method (SILAC 2-plex, arginine and lysine residue modification 13C +6.020 Da) were included in the search.

1.6 Isolation, Activation and In-Situ Hybridisation of Platelets and Neutrophils

1.6.1 Isolation of Washed Platelets from Whole Blood

Whole blood was collected from the antecubital fossa of a healthy male volunteer directly into 2 8.4ml Vacutainer tubes containing Acid/Citrate/Dextrose (ACD) buffer (Beckton Dickinson, Catalogue No. 364606). PRP was isolated by centrifugation at 200 x *g* for 15 minutes without brakes. Supernatant PRP was transferred from each tube using Pasteur pipettes to 2 fresh 15ml Falcon tubes, and centrifuged further at 1000 x *g* to pellet platelets. Supernatant plasma was

removed and discarded. The platelet pellets were resuspended in 5mls of modified Tyrode's-HEPES buffer (NaCl, 134 mM; KCl, 2.9 mM; Na_2HPO_4, 0.34 mM; $NaHCO_3$, 12 mM; HEPES, 20 mM; $MgCl_2$, 1 mM), then stored at 37°C prior to mixing with neutrophils.

1.6.2 **Isolation of Neutrophils from Whole Blood**

Neutrophils were isolated from whole blood using a two-step gradient isolation technique followed by a red cell lysis step. 40mls of blood were collected from the antecubital fossa of the same volunteer, into 4 10ml EDTA Vacutainer tubes (Beckton Dickinson, Catalogue No. 366643). 15mls of room temperature Histopaque 1077 (Sigma, catalogue number 10771-500ML) was placed in 2 50ml Falcon tubes, and 20mls of whole blood in EDTA carefully added on top, running the blood down the side of the Falcon tube using a blunt filling needle (Beckton Dickinson, Catalogue number 305180). Falcon tubes were centrifuged at room temperature, at 385 x *g* with no brakes, for 30 minutes. The resulting supernatant, composed of platelets and mononuclear cells, was carefully removed using a Pasteur pipette and discarded. The remaining pellet of red cell and neutrophils was resuspended in an equal volume of HBSS without calcium or magnesium. The resulting diluted cell mixture was then mixed with an equal volume of 2% Dextran (prepared by mixing 1g of Dextran, Sigma-Aldrich D8906-50G, with 50ml of HBSS at room temperature, vortexing for 5 minutes and incubating at 37°C for an hour). The Dextran and cell mixture was left standing upright for 20 minutes at room temperature to allow red cells to sediment. The resulting supernatant was removed and placed in fresh 50ml Falcon tubes, and centrifuged at 240 x *g* for 5 minutes with acceleration and deceleration at the medium setting. The resulting supernatant was removed and discarded, and the remaining pellet resuspended in 5mls of red cell lysis buffer (NH_4Cl 1.66 g, $NaHCO_3$ 200 mg, EDTA 7.4 mg in 100 ml ddH_2O) at 37°C for 3 minutes. Then, 47mls of HBSS was added to neutralise the lysis buffer, and the Falcon tubes again centrifuged at 240 x *g* for 5 minutes. On observing the successful lysis of red cells through the demonstration of a white pellet, cells were resuspended to a concentration of 1 million per ml in HBSS.

1.6.3 **Combination and Activation of Platelets and Neutrophils**

To determine the cellular source of proteins in neutrophil and platelet mixtures, cells were plated onto microscopy slides. Each slide was divided into three sections using a grease pen, and mixtures prepared in triplicate on each slide. 20µl of washed platelet suspension was added to each section on a slide. 10µl of platelet suspension and 10µl of neutrophil suspension were

mixed on 3 sections of slide, and 100mM Calcium Chloride added to return calcium levels to physiological levels (2.5mM). Finally, 10µl of washed platelets and 10µl of neutrophil suspension were mixed in 3 slide sections, calcium chloride was supplemented to final concentration of 2.5mM and then TRAP-6 was added to a concentration of 90nM. All slides were incubated at 37°C, then supplemented with formaldehyde to a final concentration of 4% and allowed to fix for 10 minutes. Slides were then washed three times in ice-cold PBS and prepared for fluorescence immunohistochemistry.

1.6.4 **In-Situ Hybridisation of Platelet/Neutrophil Mixtures**

Slides were incubated in 0.1% Triton X-100 (Sigma Aldrich, catalogue number T8787) in PBS for 20 minutes at room temperature, then washed with PBS-Tween (PBS-T, 0.05% Tween 20, Sigma Aldrich Catalogue Number P1379-500ML) three times. Cell-containing regions were marked out with a wax marker pen and then blocked with 5% bovine serum albumin in PBS-T for 1 hour at room temperature. The blocking solution was gently tapped off. Antibodies for CD41 (Abcam, cat: ab11024) and S100A8 (Abcam, cat: ab92331) were diluted (1 in 200) in blocking solution. Antibody dilutions were applied to slides and incubated overnight at 4°C in a humidified slide chamber. Isotope IgGs were used in the same dilutions as negative controls. Slides were then washed with PBS-T for five minutes, three times. Secondary antibodies (Donkey anti-Mouse IgG (H+L) Alexa 647, ThermoFisher Cat: A31571 and Donkey anti-Rabbit IgG (H+L) Alexa 594, ThermoFisher Cat: A21207) were diluted 1µl in 1000µl of blocking solution. Slides were incubated in secondary antibody dilutions for 1 hour at room temperature, then washed three times for five minutes each with PBS-T. Slides were incubated with 4′,6-diamidino-2-phenylindole (DAPI) for 30 minutes at room temperature, then washed again with PBS-T as before. Slides were mounted with mounting medium, and images taken with a Nikon Spinning Disc Confocal Microscope using a 60x oil objective.

2 SUPPLEMENTARY FIGURES

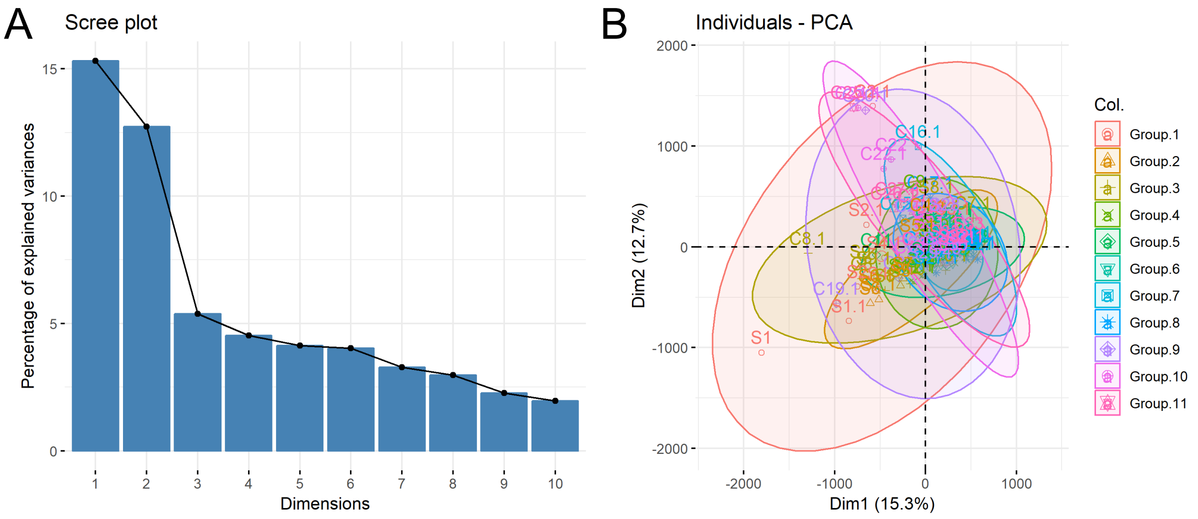

Figure SI: **Principle component analysis of TMT-groups in the TMT-tagged LC-MS/MS analysis of 110 platelet pellets during myocardial infarction. (A)** Scree plot to identify the first two principle components explaining 15.3% and 12.7% of variance. **(B).** Plot of individual samples confirms overlap of each of 11 TMT groups (sample labelled "C" from CABG group, "S" from STEMI group, numerical value as individual identifier, suffix ".1" denotes day 3 sample, absence of ".1" denotes day 0 sample").

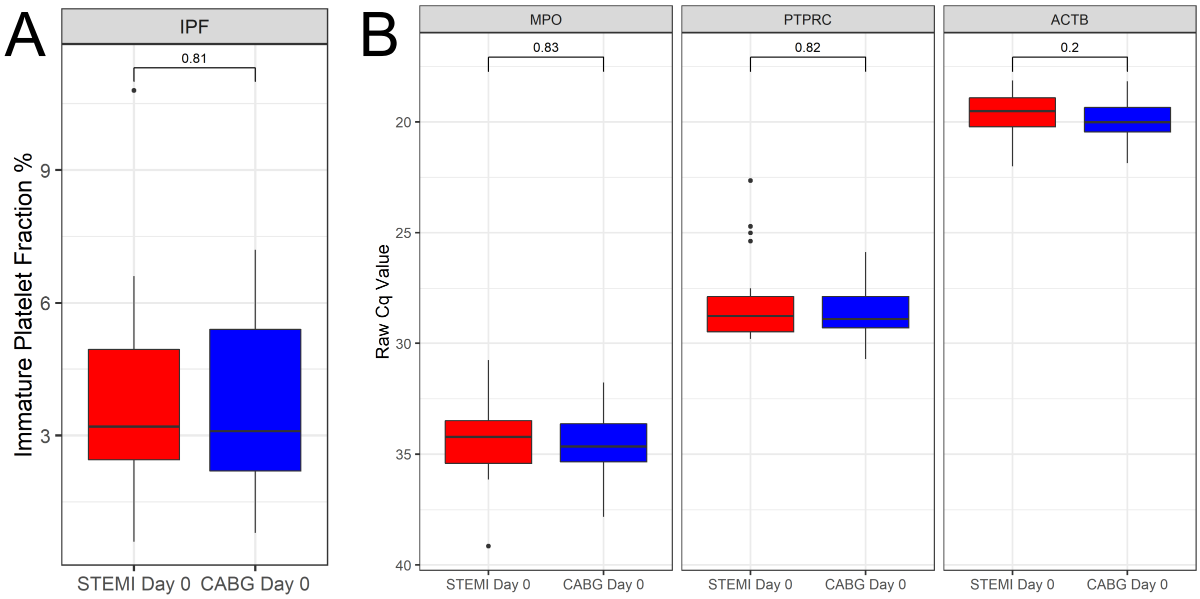

Figure SII: **RNA analysis of platelet pellet in a clinical cohort with acute and stable CVD. (A)** Immature platelet fraction (IPF), a marker of platelet RNA content was not significantly altered between groups at the time of STEMI or before CABG. **(B)** Likewise, raw Cq measurements of myeloperoxidase (MPO) and PTPRC (CD45), canonical leukocyte markers, were not differentially expressed. ACTB was used as housekeeping normaliser.

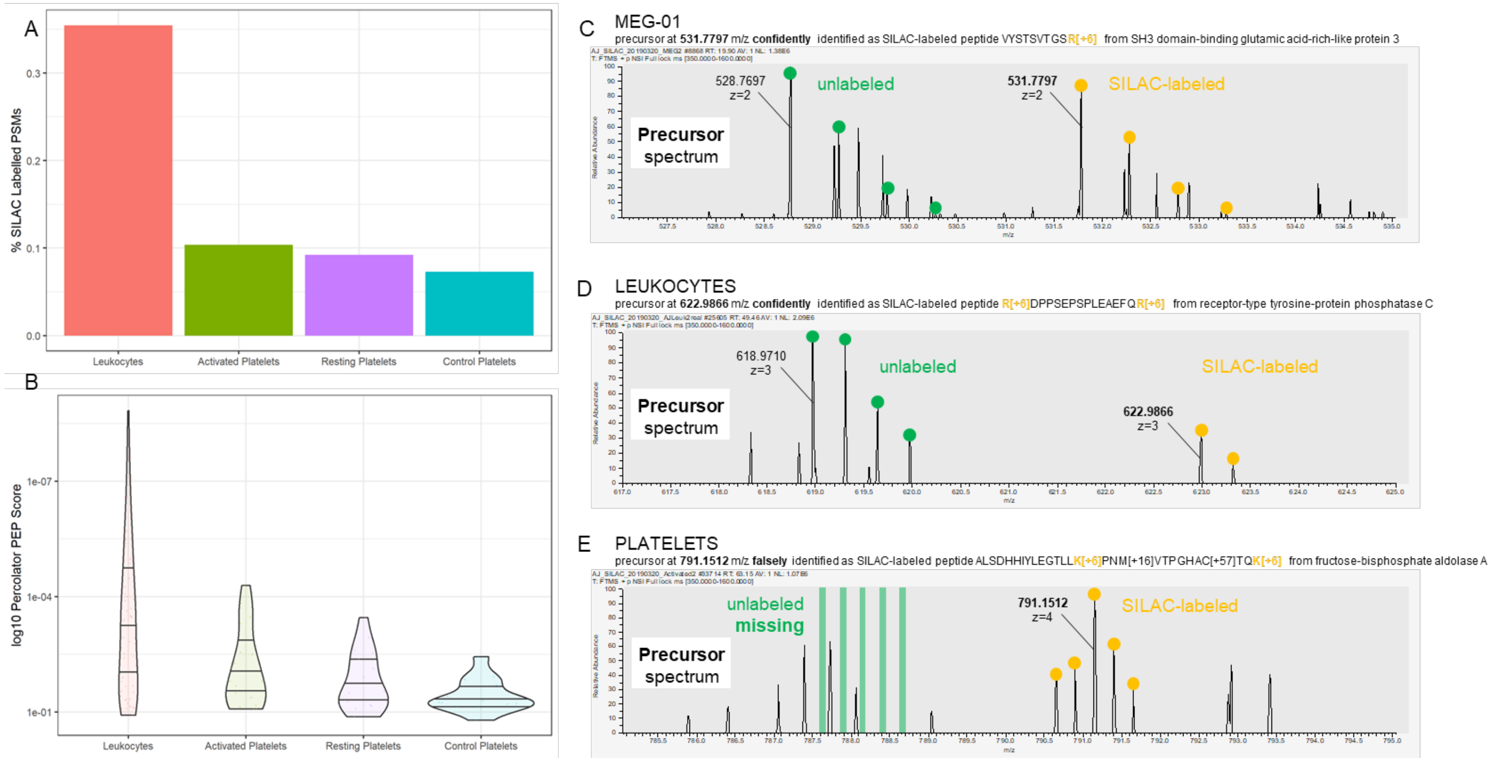

Figure SIII: **Very low levels of protein synthesis in platelets. (A) Proportion of heavy-labelled peptides in circulating blood cells after incubaltion in SILAC medium.** Automated count of peptides generated from leukocytes and platelets incubated for 24 hours in SILAC medium identified significantly more heavy-labelled peptide in leukocytes samples (n=2) than in activated (n=5) or resting platelets (n=5), which demonstrated similar synthetic potential to control platelets (n= 5) lysed after 1 hour's incubation. **(B) Automated analysis of confidence of peptide identification (Percolator PEP Score)** found significantly higher confidence of heavy-labelled peptide identification in leukocytes than in activated, resting or control platelets, which all had comparable, low confidence. **False identification of SILAC-labeled peptides in platelets by Mascot search engine.** (**C**) Precursor mass spectrum from analysis of SILAC-labeled MEG-01 digest shows SILAC-labeled (**yellow**) and unlabeled (**green**) precursor isotope distributions of peptide VYSTSVTGSR. Precursor at 531.7797 m/z was auto-selected for fragmentation using higher-energy C-trap dissociation (HCD). (**D**) Precursor mass spectrum from analysis of leukocyte digest shows SILAC-labeled (**yellow**) and unlabeled (**green**) precursor isotope distributions of peptide RDPPSEPSPLEAEFQR. Precursor at 622.9866 m/z was fragmented using HCD. (**E**) Precursor mass spectrum from analysis of TRAP-6-activated, SILAC-labeled platelet digest shows SILAC-labeled (**yellow**) precursor isotope distribution of a peptide putatively identified as ALSDHHIYLEGTLLKPNMVTPGHACTQK with a corresponding unlabeled precursor isotope distribution being absent (**green** bars).

A

#1	a⁺	b⁺	Seq.	y⁺	#2
1			**V**		10
2			**Y**		9
3			**S**		8
4			**T**		7
5			**S**		6
6			**V**		5
7			**T**		4
8			**G**		3
9			**S**		2
10			**R[+6]**		1

B

#1	a⁺	a²⁺	b⁺	b²⁺	Seq.	y²⁺	#2
1					**A**		28
2					**L**		27
3					**S**		26
4					**D**		25
5					**H**		24
6					**H**		23
7					**I**		22
8					**Y**		21
9					**L**		20
10					**E**		19
11					**G**		18
12					**T**		17
13					**L**		16
14					**L**		15
15					**K[+6]**		14
16					**P**		13
17					**N**		12
18					**M[+16]**		11
19					**V**		10
20					**T**		9
21					**P**		8
22					**G**		7
23					**H**		6
24					**A**		5
25					**C[+57]**		4
26					**T**		3
27					**Q**		2
28					**K[+6]**		1

Figure SIV: **Simplified ions tables of two peptides identified by Mascot.** (**A**) Peptide VYSTSVTGSR[+6] identified in MEG-01 analysis, (**B**) peptide ALSDHHIYLEGTLLK[+6]PNM[+16]VTPGHAC[+57]TQK[+6] identified in activated platelet analysis. Coloured rectangles show N-terminal (**red**) and C-terminal (**blue**) peptide fragment ions that were matched to theoretical fragment ions. Numbers in square brackets indicate the approximate mass shift in Dalton caused by modification of the respective residue. Ions tables correspond to the fragment spectra shown in **Supplementary Figure III**.

3 MAJOR RESOURCES TABLE

In order to allow validation and replication of experiments, all essential research materials listed in the Methods should be included in the Major Resources Table below. Authors are encouraged to use public repositories for protocols, data, code, and other materials and provide persistent identifiers and/or links to repositories when available. Authors may add or delete rows as needed.

Animals (in vivo studies)

Species	Vendor or Source	Background Strain	Sex	Persistent ID / URL
NA				

Genetically Modified Animals

	Species	Vendor or Source	Background Strain	Other Information	Persistent ID / URL
**Parent - Male**	NA				
**Parent - Female**					

Antibodies

Target antigen	Vendor or Source	Catalog #	Working concentration	Lot # (preferred but not required)	Persistent ID / URL
CD41	Abcam	ab11024	1:200		https://www.abcam.com/cd41-antibody-co35e4-ab23615.html
S100A8	Abcam	ab92331	1:200		https://www.abcam.com/s100a9-antibody-epr3555-ab92507.html
Donkey anti-Mouse	ThermoFisher	A31571	1:1000		https://www.thermofisher.com/antibody/product/Donkey-anti-Mouse-IgG-H-L-Highly-Cross-Adsorbed-Secondary-Antibody-Polyclonal/A-31571

IgG (H+L) Alexa 647					
Donkey anti-Rabbit IgG (H+L) Alexa 594	ThermoFisher	A21207	1:1000		https://www.thermofisher.com/antibody/product/Donkey-anti-Mouse-IgG-H-L-Highly-Cross-Adsorbed-Secondary-Antibody-Polyclonal/A-31571

DNA/cDNA Clones

Clone Name	Sequence	Source / Repository	Persistent ID / URL
NA			

Cultured Cells

Name	Vendor or Source	Sex (F, M, or unknown)	Persistent ID / URL
MEG-01	ATCC	male	https://www.lgcstandards-atcc.org/products/all/CRL-2021.aspx?geo_country=gb

Data & Code Availability

Description	Source / Repository	Persistent ID / URL

Other

Description	Source / Repository	Persistent ID / URL
Prostacyclin	Tocris Bioscience, 2989	https://www.tocris.com/products/epoprostenol_2989
Cell Lysis Buffer	Cell Signalling Technology, 9803	https://www.cellsignal.co.uk/products/buffers-dyes/cell-lysis-buffer-10x/9803
Protease inhibitor (PhosSTOP)	Merck, 4906845001	https://www.sigmaaldrich.com/catalog/product/roche/phossro?lang=en&region=GB
Qiazol	Qiagen, 79306	https://www.qiagen.com/gb/products/discovery-and-translational-research/lab-essentials/buffers-reagents/qiazol-lysis-reagent/#orderinginformation
Trypsin/LysC	Promega, V5072	https://www.promega.co.uk/products/mass-spectrometry/proteases-and-surfactants/trypsin_lys_c-mix_-mass-spec-grade/?catNum=V5071

QIAGen miRNAEasy mini kit	Qiagen, 217004	https://www.qiagen.com/gb/products/discovery-and-translational-research/dna-rna-purification/rna-purification/total-rna/rneasy-mini-kit/?clear=true#orderinginformation
Cel-miR-39-3p	Qiagen, 219600	https://www.qiagen.com/gb/products/discovery-and-translational-research/pcr-qpcr-dpcr/qpcr-assays-and-instruments/mirna-qpcr-assay-and-panels/miscript-mirna-mimics/?clear=true#orderinginformation
MS2 RNA	Roche, 10165948001	https://www.sigmaaldrich.com/catalog/product/roche/10165948001?lang=en&region=GB
Heparinase I	Sigma, 9025-39-2	https://www.sigmaaldrich.com/catalog/product/sigma/h2519?lang=en&region=GB&gclid=Cj0KCQiApY6BBhCsARIsAOI_GjZbABksOeHtXNacHNqn-WzNLvm5r8Fm4K8qRG2NsAd7Gnfy_gutf7gaAu43EALw_wcB
Ribo Lock RNase Inhibitor	Thermo Fisher,EO0382	https://www.thermofisher.com/order/catalog/product/EO0382?gclid=Cj0KCQiApY6BBhCsARIsAOI_Gja5oeeSkGKj34IFifMxdXlieOUHRnWPzY6VXVRirv4C5Keaav7xQ3gaAirsEALw_wcB&ef_id=Cj0KCQiApY6BBhCsARIsAOI_Gja5oeeSkGKj34IFifMxdXlieOUHRnWPzY6VXVRirv4C5Keaav7xQ3gaAirsEALw_wcB:G:s&s_kwcid=AL!3652!3!482647966444!p!!g!!ribolock&cid=bid_mol_clo_r01_co_cp1358_pjt0000_bid00000_0se_gaw_bt_pur_con#/EO0382?gclid=Cj0KCQiApY6BBhCsARIsAOI_Gja5oeeSkGKj34IFifMxdXlieOUHRnWPzY6VXVRirv4C5Keaav7xQ3gaAirsEALw_wcB&ef_id=Cj0KCQiApY6BBhCsARIsAOI_Gja5oeeSkGKj34IFifMxdXlieOUHRnWPzY6VXVRirv4C5Keaav7xQ3gaAirsEALw_wcB:G:s&s_kwcid=AL!3652!3!482647966444!p!!g!!ribolock&cid=bid_mol_clo_r01_co_cp1358_pjt0000_bid00000_0se_gaw_bt_pur_con
RPMI cell culture medium	Thermo FIsher, A2494201	https://www.thermofisher.com/order/catalog/product/A2494201?uk&en#/A2494201?uk&en
$^{13}C_6$-labelled heavy isotope lysine	Thermo Fisher, 89988	https://www.thermofisher.com/order/catalog/product/89988?uk&en#/89988?uk&en

$^{13}C_6$-labelled heavy isotope arginine	Thermo Fisher, 88210	https://www.thermofisher.com/order/catalog/product/88210?uk&en#/88210?uk&en
Hanks' Balanced Saline Solution	Sigma, H6648	https://www.sigmaaldrich.com/catalog/product/sigma/h6648?lang=en&region=GB
8.4ml Vacutainer tubes containing Acid/Citrate/Dextrose (ACD) buffer	Beckton, Dickinson, 364606	https://www.bd.com/en-us/offerings/capabilities/specimen-collection/blood-specimen-collection/venous-collection/bd-vacutainer-blood-collection-tubes/view-specialty-tubes
10ml EDTA Vacutainer tubes	Beckton, Dickinson, 366643	https://www.bd.com/en-us/offerings/capabilities/specimen-collection/blood-specimen-collection/venous-collection/bd-vacutainer-blood-collection-tubes/view-hematology-tubes
Histopaque 1077	Sigma, 10771	https://www.sigmaaldrich.com/catalog/product/sigma/10771?lang=en&region=GB

Dextran	Sigma, D8906-50G	https://www.sigmaaldrich.com/catalog/substance/dextransulfatesodiumsaltfromleuconostocspp12345901118111?lang=en&region=GB&gclid=Cj0KCQiApY6BBhCsARIsAOI_GjbrOTKaZsjtiejy6KSCBd_nNVGhAS_3V6PlzD2Ncsn_woxOTmKMDOYaAkiEEALw_wcB
Triton X-100	Sigma, T8787	https://www.sigmaaldrich.com/catalog/product/sigma/t8787?lang=en&region=GB&gclid=Cj0KCQiApY6BBhCsARIsAOI_GjYqJ4XXpD79CxESZmF8XFaiXlu3YCqAmlmX7LexfgnkhaqdYxNhdu0aAqItEALw_wcB
PBS-Tween	Sigma, P1379-500ML	https://www.sigmaaldrich.com/catalog/search?term=P1379-500ML&interface=ALL&N=0+&mode=partialmax&lang=en&region=GB&focus=product
S100A8/A9 DuoSet ELISA Development kits and Duoset Ancillary Reagent Kit 2	R&D, DY5578	https://www.rndsystems.com/products/human-s100a9-duoset-elisa_dy5578
