# Supplementary figures and images for: Neutrophil-Derived Protein S100A8/A9 Alters the Platelet Proteome in Acute Myocardial Infarction and Is Associated With Changes in Platelet Reactivity
Source: Arterioscler Thromb Vasc Biol. 2021 Nov 23;42(1):49–62. doi: 10.1161/ATVBAHA.121.317113 (PMC8691374; doi:10.1161/ATVBAHA.121.317113)

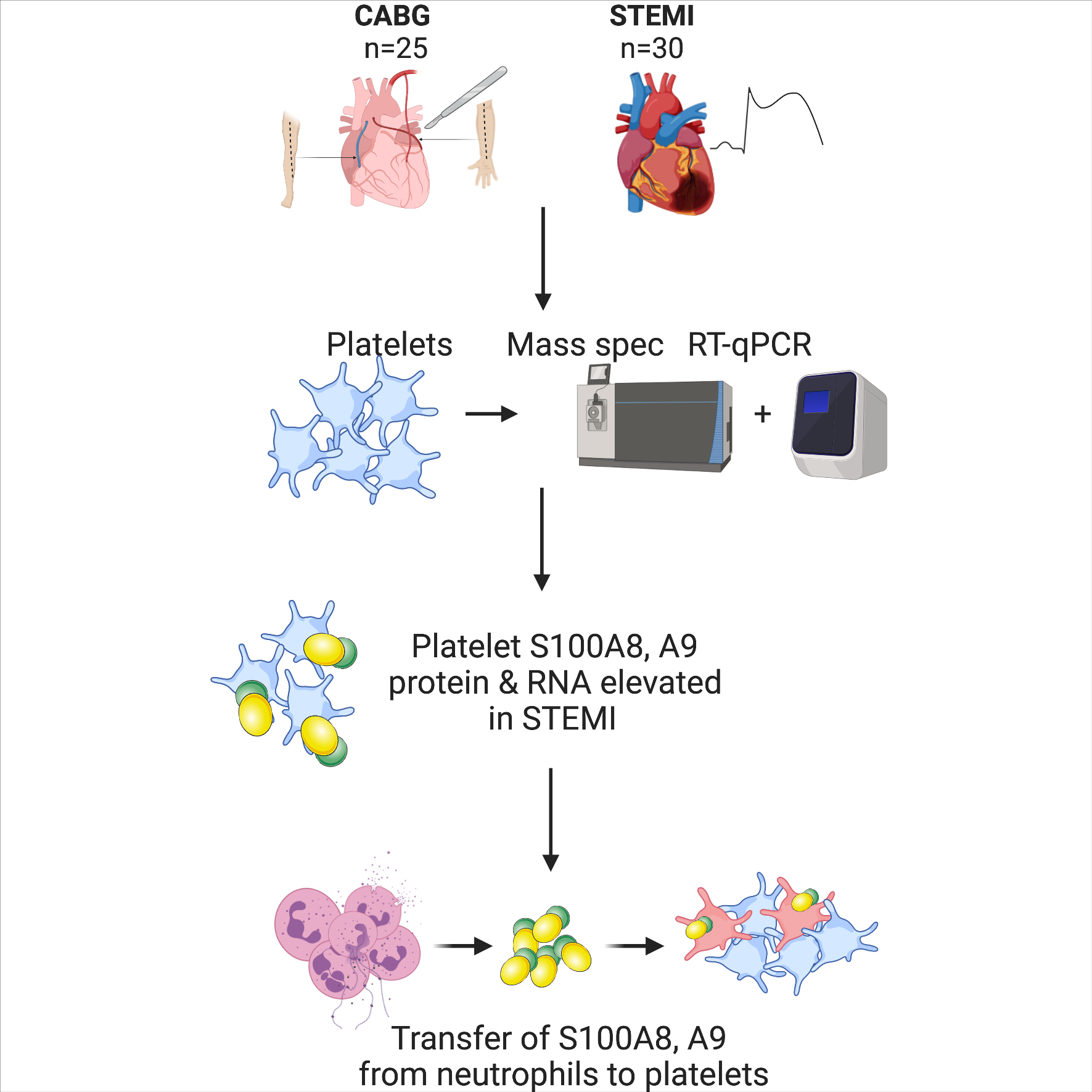

Supplement: Supplementary file 4 [file atv-42-049-s004.jpg]
